# Toll-like receptor 7 affects the pathogenesis of non-alcoholic fatty liver disease

**DOI:** 10.1038/srep27849

**Published:** 2016-06-09

**Authors:** Sokho Kim, Surim Park, Bumseok Kim, Jungkee Kwon

**Affiliations:** 1 Department of Laboratory Animal Medicine, Chonbuk National University, 79 Gobongro, Iksan, 54596, Republic of Korea; 2 Laboratory of Pathology, College of Veterinary Medicine, Chonbuk National University, 79 Gobongro, Iksan, 54596, Republic of Korea

## Abstract

Recently, a possible link between toll-like receptor 7 (TLR7) and liver disease was suggested, although it was limited to fibrosis. Based on this report, we investigated whether TLR7 has a pivotal role in non-alcoholic fatty liver disease (NAFLD). The TLR7 signaling pathway, which is activated by imiquimod (TLR7 ligand) naturally, induced autophagy and released insulin-like growth factor 1 (IGF-1) into medium from hepatocytes. Lipid accumulation induced by unsaturated fatty acid (UFA; arachidonic acid:oleic acid = 1:1) in hepatocytes, was attenuated in TLR7 and autophagy activation. Interestingly, TLR7 activation attenuated UFA-induced lipid peroxidation products, such as malondialdehyde (MDA) and 4-Hydroxy-2-Nonenal (4-HNE). To clarify a possible pathway between TLR7 and lipid peroxidation, we treated hepatocytes with MDA and 4-HNE. MDA and 4-HNE induced 2-folds lipid accumulation in UFA-treated hepatocytes via blockade of the TLR7 signaling pathway’s IGF-1 release compared to only UFA-treated hepatocytes. *In vivo* experiments carried out with TLR7 knockout mice produced results consistent with *in vitro* experiments. In conclusion, TLR7 prevents progression of NAFLD via induced autophagy and released IGF-1 from liver. These findings suggest a new therapeutic strategy for the treatment of NAFLD.

Non-alcoholic fatty liver disease (NAFLD) is regarded as the major symptom in the liver in patients with metabolic syndrome, and is most common serious threat to public health worldwide^1^,^2^,^3^,^4^. A recent epidemiologic study on NAFLD reported that nearly 50% of patients also have type 2 diabetes, and 100% also have pathological obesity^5^. NAFLD comprises a range of hepatic disease, which begins as lipid accumulation in the liver, also known as hepatic steatosis. This massive lipid accumulation perturbs liver function by various possible mechanisms and may progress to non-alcoholic steatohepatitis (NASH), which is combined with inflammation and fibrosis^6^. Hepatic cirrhosis, which is the end stage of chronic liver disease, is caused by the progression of fibrosis. Within the next decade, up to 20% of patients suffering from NASH will progress to liver cirrhosis, and 10% of patients will die from a condition related to chronic liver disease^7^. Although a clear mechanism by which NAFLD accelerates the progression of chronic liver disease is poorly understood, a “two-hit” hypothesis has been suggested as a possible mechanism of pathological change in chronic liver disease^6^. This “two-hit” hypothesis was suggested to explain a common mechanism that leads to steatohepatitis and its sequelae of cirrhosis or liver failure. Briefly, simple hepatic steatosis caused by lipid accumulation represents the “first hit” in pathologic progression and after this stage, the “second hit,” which includes oxidative stress, lipid peroxidation, inflammatory cytokines, hormone dysfunction, and mitochondrial dysfunction, is required for progressive forms of chronic liver disease, such as NASH and cirrhosis^8^. Artificial deletion of insulin-like growth factor 1 (IGF-1), which regulates lifespan and metabolism in the liver^9^, activates autophagy, and performs “house-keeping” actions under physiological and pathophysiological conditions^10^ improved NAFLD. Nevertheless, the exact underlying mechanisms of molecular change and the triggers which accelerate pathologic change remain unclear.

Toll-like receptors (TLRs), a family which consists of approximately 13 pattern recognition receptors, are the most important innate immune response factors involved in host defense against foreign pathogens. Innate adaptive immune responses induced by various cytokines follow the interaction of TLRs with each specific ligand^11^. Recent studies about liver diseases and TLRs have suggested various possible mechanisms related to pathophysiological change due to immune responses^12^,^13^,^14^. TLR7 is a commonly identified that response to viruses, bacteria, and other TLR7 agonists^15^. Although the role of TLR7 signaling in liver fibrosis has been demonstrated^14^, the role of TLR7 signaling in the progression of NAFLD has not been elucidated. Therefore, we investigated TLR7 and the “two-hit” hypothesis to provide mechanistic insight into this complex and dynamic disease.

The aim of the present study was to demonstrate whether TLR7 is required for progressive NAFLD. Moreover, we clarified the role of autophagy, IGF-1, and lipid peroxidation products such as malondialdehyde (MDA) and 4-Hydroxy-2-Nonenal (4-HNE), which are associated with TLR7, at both the cellular and tissue levels under NAFLD. This study was divided into two stages, consistent with research purposes. First, we explored the relationship between TLR7 and autophagy and the relationship between TLR7 and IGF-1 secretion. Second, we elucidated whether MDA and 4-HNE could exert deleterious effects on TLR7 levels and clarified the possible mechanisms underlying the role of TLR7 in preventing progressive NAFLD. We confirmed these *in vitro* results using an *in vivo* mouse model of NAFLD.

## Materials and Methods

### Chemicals

Imiquimod was purchased from InvivoGen (San Diego, CA, USA). Rapamycin, 3-MA, and 4-HNE were purchased from Santa Cruz Biotech (Dallas, TX, USA). Doxycycline (DOX) was purchased from Clontech (Mountain View, CA, USA). MDA, arachidonic acid, oleic acid, and other standard reagents were purchased from Sigma (St. Louis, MO, USA). Primary antibodies for TLR7, Myd88, LC3A/B, IGF-1, and β-actin were purchased from Cell Signaling Technology (Beverly, MA, USA). Secondary antibodies (i.e., anti-rabbit, anti-goat, or anti-mouse IgG antibody conjugated with horseradish peroxidase; anti-rabbit IgG antibody conjugated with Alexa Fluor 488) were obtained from Millipore (Temecula, CA, USA). Hoechst staining kit was purchased from Invitrogen (Carlsbad, CA, USA). All other chemicals and reagents were of analytic grade.

### Preparation of unsaturated fatty acids mixtures (UFAs)

We prepared unsaturated fatty acids (UFAs) to investigate NAFLD progression, as they may negatively regulate NAFLD^16^ and could be involved in liver diseases^17^. UFA-induced lipid accumulation in the liver strongly affects histological change in hepatic steatosis. Among these UFAs, arachidonic acid is omega-6 polyunsaturated fatty acid, and it is involved in the synthesis of lipid peroxidation products such as MDA and 4-HNE during oxidative stress^18^. Oleic acid, which is an omega-9 monounsaturated fatty acid, leads to lipid accumulation in an *in vitro* hepatocyte model^19^. Thus, the UFAs for this study were mixed from arachidonic acid and oleic acid (molar ratio 1:1).

### Animals and *in vivo* experimental design

8-week old TLR7-deficient mice (TLR7KO) were used in these studies (25–30 g body weight). Dr. Akira (Osaka University, Suita, Japan) kindly provided the TLR7KO mice on a C57BL/6 background. Normal C57BL/6 mice were purchased from the SLC (Hamamatsu, Japan) and used as a wild type control. The mice were maintained in microisolator cages under pathogen-free conditions on a 12-h/12-h light/dark cycle, and housed under controlled temperature (23 ± 3°C, mean ± range) and humidity (about 60%) conditions. All animals were cared for in accordance with institutional ethical guidelines for the care and use of experimental animals at Chonbuk National University. The animal facility of the Chonbuk National University is fully accredited by the National Association of Laboratory Animal Care. All experimental protocols using animal were approved by the animal facility of the Chonbuk National University. To establish NAFLD conditions in experimental mice, UFAs were included in a modified mouse diet based on the normal research diet (Research Diets, New Brunswick, NJ, USA), and contained 45% of its calories from fat. Detailed components of the experimental diet are described in the supporting information (Table S1). Each group consisted of 6 mice, and a normal control group was fed a normal control diet, while NAFLD induction groups were fed a UFAs diet for eight weeks. During the experimental period, mice in specific experimental groups were intra-peritoneally administered imiquimod (0.1 mg/kg), rapamycin (2 mg/kg), MDA (0.4 mg/kg), and/or 4-HNE (0.8 mg/kg), in 0.1 ml saline twice a week. At the end of the experimental period, food was withheld for 12 h and the mice were anesthetized with ether. Blood samples were taken from the inferior vena cava to analyze serum biomarkers. Subsequently, cardiac punctures were performed and the mice were perfused with cold physiological saline. The livers were then harvested, photographed, and processed. Livers were processed for histology, immunoblotting, and other analyses; samples were stored at −80 °C until analysis.

### Cell culture and *in vitro* experimental design

Hepatocytes were isolated from C57BL/6 and TLR7KO mice as previously described^20^. Cells were cultured at 37 °C under a humidified, 5% CO_2_ atmosphere in Dulbecco’s Modified Eagle’s Medium with 10% fetal bovine serum (FBS), 100 U/ml penicillin, and 100 μg/ml streptomycin. After cell stabilization, hepatocytes were serum starved for 6 h and then treated with imiquimod (10 μg/ml), rapamycin (50 μM), 3-Methyladenine (3-MA; 10 mM), MDA (40 μM), 4-HNE (40 μM), TLR7 siRNA, and Myd88 siRNA, with or without scrambled siRNA in the presence or absence of UFAs (10 mM) for 72 h. Additionally, the HepG2 Tet-On^®^ Advanced Cell Line transfected with IGF-1-GFP (Tet-On cells) was purchased from Clontech (CA, USA) and used for examining the release of intrinsic inducible IGF-1. IGF-1-GFP that was induced by DOX (1 μg/ml) treatment for 72 h was incubated with a corresponding agent such as rapamycin, imiquimod, 3-MA, or TLR7 siRNA, with or without scrambled siRNA.

### Small interfering RNA transfection

Cells were plated in the indicated plastic ware until reaching confluence and were then starved for 6 h. The DharmaFECT 1 small interfering RNA (siRNA) Transfection Reagent (Dharmacon, Denver, CO, USA) was used to transfect the cells with 50 nM TLR7 siRNA, Myd88 siRNA, or scrambled siRNA oligonucleotides (Dharmacon) according to the manufacturer’s instructions and as reported previously.

### Transmission electron microscopy (TEM)

The cells and liver tissue were fixed via circulation with a fixation buffer composed of 2% paraformaldehyde (PFA), 2.5% glutaraldehyde (Sigma, St. Louis, MO, USA), and 3 mM calcium chloride in 0.1 M sodium cacodylate buffer. Samples were placed in fixation buffer on ice for 2 h, and then were washed 6 times with buffer consisting of 0.1 M sodium cacodylate and 3 mM calcium chloride on ice. The samples were vibratomed with a Leica VT 1000s vibratome at 80 μm and then postfixed with 1% osmium tetroxide, 0.8% potassium ferrocyanide, and 3 mM calcium chloride in 0.1 M sodium cacodylate for 1 h. Samples were then washed 3 times with ice-cold distilled water, stained *en bloc* with 2% uranyl acetate at 4 °C overnight, dehydrated through graded ethanol solutions, and embedded in Durcupan ACM resin (Sigma). Ultrathin 80-nm sections were made with a Leica Ultracut UCT ultramicrotome, and sections were poststained with uranyl acetate and lead salts prior to imaging using a HITACHI H-7650 TEM operated at 80 kV (Indicated magnification: X25,000).

### Fluorescence analyses

Hepatocytes were fixed with 2% PFA, washed three times with PBS, permeabilized with 0.1% Triton X-100 in PBS for 10 min, and then washed three times with PBS. Fixed cells were incubated for 30 min with 1% BSA in PBS. Cells were incubated overnight with diluted LC3A/B primary antibody (1:1,000 dilution). After rinsing in PBS, these cells were incubated for 2 h with diluted secondary antibody (anti-rabbit Alexa Fluor 488) and washed with PBS. Tet-On cells were also fixed with the corresponding procedure but no immunostaining, after which we collected the supernatant medium. Subsequently, nuclear staining was performed using Hoechst (1:10,000 dilution) for 5 min. After Hoechst staining, images were obtained by fluorescence microscopy on a scope A1 (Indicated magnification: X200; Carl Zeiss, Jena, Germany). Additionally, we quantified the reactive fluorescence units (RFU) of Tet-On cells in PBS in collected supernatant medium from Tet-On cells using an excitation wavelength of 490 nm and an emission wavelength of 525 nm.

### Oil Red O stain

Lipid accumulation was analyzed using an Oil Red O staining kit as described in the manufacturer’s protocol (Lifeline Cell Technology, Carlsbad, CA, USA). Oil Red O-stained cells were imaged with an Observer A1 microscope (Carl Zeiss) at X200 magnification. After image acquisition, accumulated intracellular Oil Red O dye was eluted with isopropanol and quantified by determining its optical absorbance at 540 nm using a PowerWave2 multiplate reader spectrophotometer (Bio-Tek Instruments, Winooski, VT, USA).

### Intracellular reactive oxygen species (ROS) assay

The level of intracellular ROS was quantified by fluorescence using 2′,7′-dichlorodihydrofluorescindiacetate (DCF-DA; Invitrogen). Cells were grown on 48-well plates and incubated with the corresponding treatment conditions for 3 h. After the incubation period, cells were washed with phosphate-buffered saline (PBS) and stained with DCF-DA in PBS for 30 min in the dark. Cells were then washed twice with PBS and extracted with 0.1% Tween-20 in PBS for 10 min at 37 °C. Fluorescence was recorded using an excitation wavelength of 490 nm and an emission wavelength of 525 nm.

### Enzyme-linked immunosorbent assay (ELISA)

The content of IGF-1 in the supernatant *in vitro* and the circulating whole-body serum content of IGF-1 *in vivo* were measured by ELISA using a kit according to the manufacturer’s instructions (Abcam, Cambridge, MA, USA). The cellular content of MDA and 4-HNE was measured by ELISA using a kit according to the manufacturer’s instructions (Cell Biolabs, San Diego, CA, USA). Hepatic content of triglycerides and cholesterol was also measured by ELISA using a kit according to the manufacturer’s instructions (Cell Biolabs). Absorbance at 560 nm was measured at using a microplate reader.

### Histopathology

Dissected liver tissues were fixed in 4% PFA for 24 h. All the fixed samples were embedded in paraffin and cut into 5 micron sections. The slide sections were de-paraffinized with xylene, rehydrated to water with an alcohol series in graduated concentrations, and stained with hematoxylin and eosin (H & E). Tissue histopathology was then evaluated using a conventional light microscope (Indicated magnification: X200; Carl Zeiss). H & E-stained sample sections were imaged and the areas of the stained regions, in pixels, were quantified using the Image Pro analysis program.

### Serum analyses

All serum biochemistry tests that included aspartate aminotransferase (AST), alanine transaminase (ALT), triglyceride, and cholesterol were examined through a VetTest 8008 Chemistry Analyzer (IDEXX, Seoul, Korea).

### Immunoblotting

Total proteins from cell lysates and liver tissue lysates were subjected to sodium dodecyl sulfate polyacrylamide gel electrophoresis using 10% to 15% gels. Proteins were then electrophoretically transferred to polyvinylidene difluoride membranes (Bio-Rad Laboratories, Hercules, CA, USA). Membranes were blocked in 5% skim milk in PBS and then incubated overnight at 4 °C with primary antibodies, which were diluted 1:1,000 in 1% skim milk in PBS. Membranes were then incubated with peroxidase-conjugated anti-rabbit, anti-goat, or anti-mouse IgG antibodies (1:5,000; Millipore, Bedford, MA, USA) for 1 h. Immunoreactive bands were visualized with SuperSignal West Dura Extended Duration Substrate (Thermo Scientific, San Jose, CA, USA) and analyzed using a chemiImager analyzer system (Alpha Innotech, San Leandro, CA, USA).

### RNA preparation and real-time RT-PCR

Total RNA was isolated from cells and precipitated with Ribo EX (Geneall, Daejeon, Korea) following the manufacturer’s protocols. The mRNA was reverse transcribed to cDNA using a Maxime RT PreMix kit (Intron, Seongnam, Korea) following the manufacturer’s protocols. For real-time RT-PCR, cDNA was amplified using a Mastercycler Gradient 5331 Thermal Cycler (Eppendorf, Germany). Real-time PCR runs were monitored by measuring the fluorescence signal after each cycle with an ABI Step One Plus Sequence Detection System (Applied Biosystems, Singapore). Specific primers for each gene were designed using Primer Express software (Applied Biosystems). The following forward and reverse primers were used for real-time RT-PCR quantification (forward and reverse): 5′-AAAGCAGCCCCGCTCTATCC-3′ and 5′-CTTCTGAGTCTTGGGCATGTCA-3′ for IGF-1, and 5′-GCATGGCCTTCCGTGTTC-3′ and 5′-GATGTCATCATACTTGGCAGGTTT-3′ for glyceraldehyde-3-phosphate dehydrogenase (GAPDH), the housekeeping gene used as an internal control. All experiments were performed at least three times.

### Statistical analysis

Results are presented as means ± standard error. Data were analyzed using the Student’s *t*-test (for two groups) or one-way ANOVA, and Tukey’s test (for more than two groups). *P* values < 0.05 were considered statistically significant. All analyses were performed using the Statistical Package for Social Sciences, version 13.0, for Windows (SPSS, Inc., Chicago, IL, USA).

## Results

### TLR7 activates autophagy in hepatocytes

**Figure 1:**
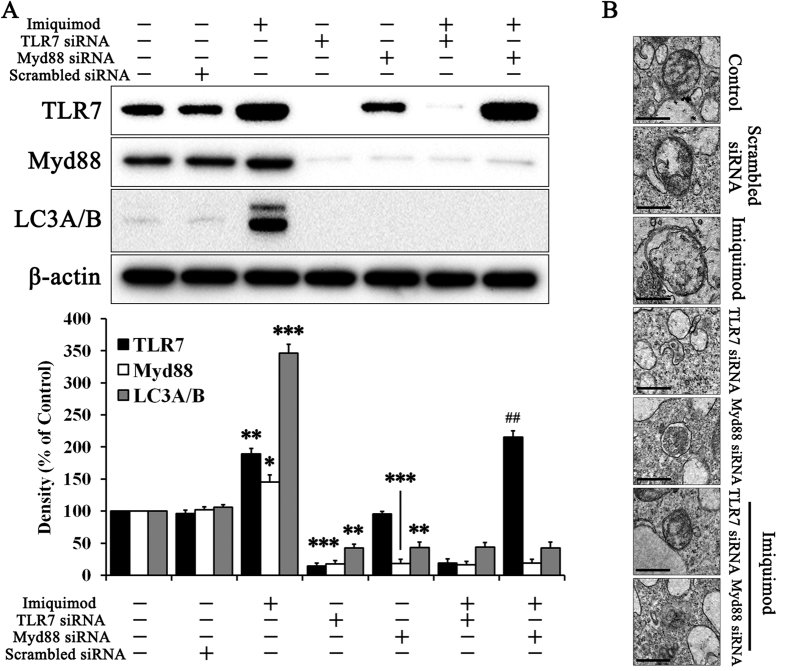
TLR7 evoked autophagy via Myd88 in hepatocytes. Hepatocytes were treated with or without 10 μg/ml imiquimod and TLR7 siRNA, Myd88 siRNA, or scrambled siRNA for 72 h and then immunoblotting was performed to evaluate (**A**) protein expression of TLR7, Myd88, LC3A/B, and β-actin. (**B**) Images of autophagosome formation were obtained from TEM in the corresponding groups. Scale bars indicate 500 nm. Data are mean and SEM values (*n* = 3). *p < 0.05 vs. untreated control. **p < 0.01 vs. untreated control. ***p < 0.001 vs. untreated control. ^##^*p* < 0.01 vs. Myd88 siRNA group.

We determined that TLR7 is induced by imiquimod, a specific TLR7 inducer that may activate autophagy in hepatocytes (Fig. 1). A previous study on TLR7 in autophagy only used immune cells^21^. As shown in Fig. 1A, imiquimod significantly increased TLR7 expression 3.5 fold compared with the non-treated control. Myd88, an immune signaling pathway intermediate that is initiated by TLR7, was also increased. LC3A/B, which serves as a marker for the autophagosome, was confirmed in the activation of autophagy^20^. Imiquimod significantly increased LC3A/B levels. Naturally, imiquimod induced significant autophagosome formation (Fig. 1B). We investigated this pathway in the absence of TLR7 and Myd88 by using siRNA methods. Only TLR7 siRNA treated group did not revealed TLR7 expression as well as Myd88, caused by deletion of TLR7 subsequently affects absence of Myd88^21^. While, Myd88 siRNA treatment did not change protein expression of TLR7, just reduced protein expression of Myd88, caused by Myd88 is downstream signaling pathway of TLR7. Thus, we thought that external treatment of TLR7 siRNA reduced protein expression of TLR7, as well as Myd88 commonly. In the absence of TLR7 or Myd88, imiquimod did not activate autophagy. Thus, the TLR7 response is essential for the downstream signaling pathway and autophagy activation in hepatocytes.

### Autophagy is activated by TLR7 release of IGF-1 from hepatocytes

**Figure 2:**
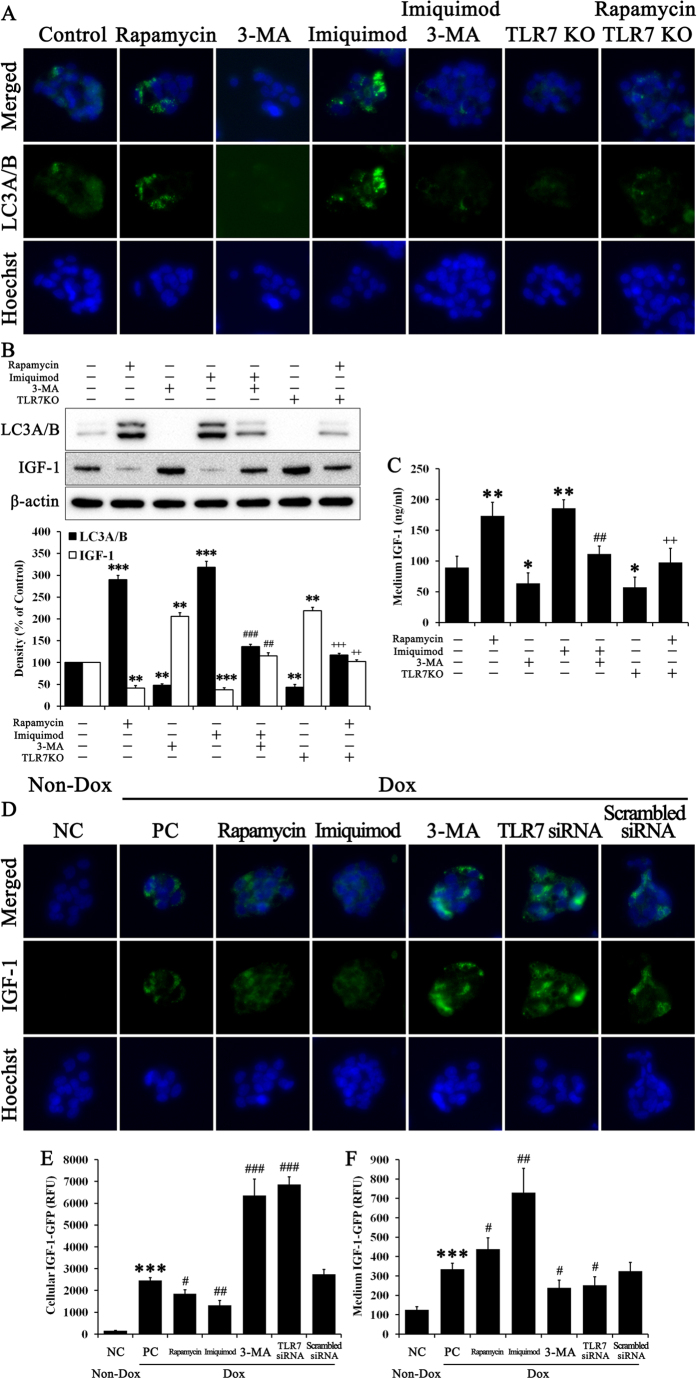
Autophagy activated by TLR7 regulates the IGF-1 balance between intracellular and extracellular fluid. Hepatocytes and TLR7KO hepatocytes were treated with or without 50 μM rapamycin, 10 mM 3-MA, and 10 μg/ml imiquimod for 72 h. We then performed (**A**) fluorescence analyses of immunostained LC3A/B and (**B**) immunoblotting to evaluate the protein expression of LC3A/B and IGF-1. (**C**) Medium from the cell supernatant was used to evaluate IGF-1 contents on ELISA. Data are mean and SEM values (*n* = 3). **p < 0.01 vs. untreated control. ***p < 0.001 vs. untreated control. ^##^*p* < 0.01 vs. 3-MA group. ^###^*p* < 0.001 vs. 3-MA group. ^++^*p* < 0.01 vs. TLR7KO group. ^+++^*p* < 0.001 vs. TLR7KO group. Tet-On cells were treated with or without 1 μg/ml DOX, 50 μM rapamycin, 10 mM 3-MA, and 10 μg/ml imiquimod and non siRNA, TLR7 siRNA, or scrambled siRNA for 72 h. DOX treatment induced intrinsic IGF-1-GFP as a fluorescence reporter and increased protein content. (**D**) Fluorescence images for GFP-tagged IGF-1 in cells and (**E**) quantitative analyses of fluorescence intensity to evaluate cellular IGF-1 content were performed. (**F**) Medium from the cell supernatant was also used to evaluate IGF-1-GFP content. The histogram was calculated by RFU. Data are mean and SEM values (*n* = 3). ***p < 0.001 vs. untreated control. ^#^*p* < 0.05 vs. PC group. ^##^*p* < 0.01 vs. PC group. ^###^*p* < 0.001 vs. PC group.

LC3A/B was visualized by fluorescence analyses (Fig. 2A). Naturally, rapamycin, an autophagy activator, increased the intensity of LC3A/B fluorescence, while 3-MA, an autophagy inhibitor, reduced the intensity of LC3A/B fluorescence in hepatocytes. Imiquimod also significantly increased LC3A/B fluorescence compared with the control, while 3-MA abrogated the effect of imiquimod on LC3A/B fluorescence. TLR7KO hepatocytes revealed a low intensity of LC3A/B fluorescence compared with the control, but rapamycin slightly increased LC3A/B fluorescence against TLR7KO-attenuated autophagy. We confirmed immunoblotting results used with corresponding experimental cell lysates. Protein expression of LC3A/B (Fig. 2B) trended with LC3A/B fluorescence (Fig. 2A). Interestingly, protein expression of IGF-1 was inversely correlated with LC3A/B expression. Rapamycin and imiquimod decreased IGF-1 when compared with controls. TLR7KO hepatocytes revealed high levels of IGF-1 expression compared to normal hepatocytes. Unlike protein expression of IGF-1 from cell lysates, secreted IGF-1 increased during rapamycin and imiquimod treatment (Fig. 2C). Moreover, TLR7KO cells had lower secreted IGF-1 than control cells. 3-MA treatment reduced IGF-1 secretion against each treatment of imiquimod and rapamycin induced it under TLR7KO conditions. Based on these results, we hypothesized that autophagy-induced IGF-1 release from cells involved TLR7. Autophagy may play a role in the transport and secretion of biomolecules^22^. To test this hypothesis, we used engineered Tet-On cells with intrinsic IGF-1 and induced HepG2 cells transfected with IGF-1-GFP (Fig. 2D–F). DOX treatment induced IGF-1-GFP expression in Tet-On cells. As shown in Fig. 2D, Tet-On cells treated with rapamycin or imiquimod revealed weak GFP fluorescence compared with positive controls (PC), while 3-MA or TLR7 siRNA strongly increased GFP fluorescence in all samples. This intensity of cellular fluorescence was quantified with a fluorescence analyzer and graphed (Fig. 2E). The result of quantitative analysis produced similar results to fluorescence imaging. Interestingly, secreted IGF-1-GFP, which was supposedly released from Tet-On cells, was inversely correlated with intracellular IGF-1-GFP (Fig. 2F). IGF-1-GFP, regarded as a reporter of intrinsic IGF-1, was supposed to transport from cells via autophagy activation. These results are important to progressive NAFLD, because IGF-1 is primarily made by the liver as an endocrine hormone to maintain lipid metabolism homeostasis. IGF-1 is essential for bodily growth and the maintenance of normal liver function. However, abnormal expression of IGF-1 in the liver may induce lipid accumulation and hepatocarcinoma^23^,^24^,^25^. Moreover, many studies show low circulating IGF-1 in NAFLD conditions^26^,^27^,^28^. We therefore suggest that our *in vitro* results may help to explain the pathology of NAFLD.

### UFAs-induced lipid accumulation was accelerated by the absence of TLR7, followed by inactivation of autophagy: “First hit” hypothesis confirmation

**Figure 3:**
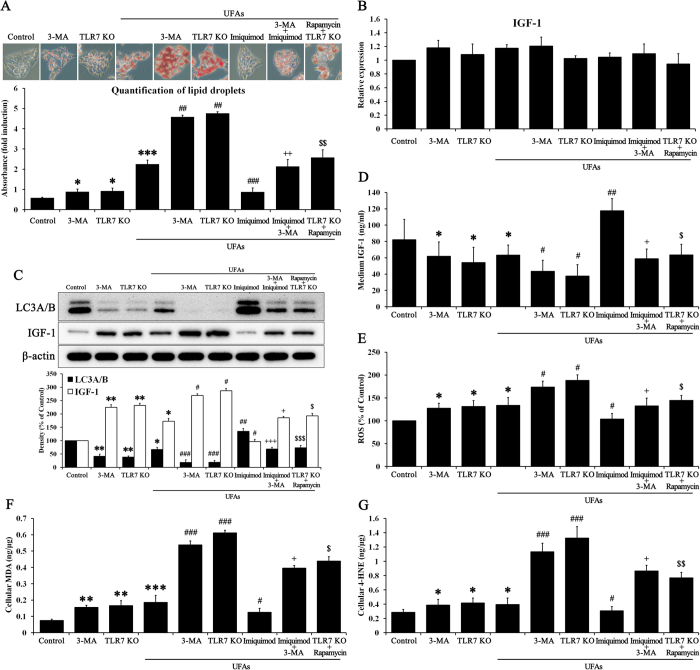
UFAs-induced lipid accumulation was prevented by TLR7, followed by autophagy activation. Hepatocytes and TLR7KO hepatocytes were treated with or without 50 μM rapamycin, 10 mM 3-MA, and 10 μg/ml imiquimod in presence or absence of 10 mM UFAs for 72 h. (**A**) Oil Red O staining was performed to evaluate lipid accumulation. The red-stained areas indicated lipid droplets, which were quantified by absorbance measurement. (**B**) Gene expression of IGF-1 in cells was examined by real-time RT-PCR. (**C**) We performed immunoblotting to evaluate the protein expression of LC3A/B and IGF-1. (**D**) Medium from the cell supernatant was used to evaluate IGF-1 contents on ELISA. (**E**) ROS production was examined in cells. (**F**) MDA production and (**G**) 4-HNE production in cells were examined by ELISA. Data are mean and SEM values (*n* = 3). *p < 0.05 vs. untreated control. **p < 0.01 vs. untreated control. ***p < 0.001 vs. untreated control. ^#^*p* < 0.05 vs. UFAs group. ^##^*p* < 0.01 vs. UFAs group. ^###^*p* < 0.001 vs. UFAs group. ^+^*p* < 0.05 vs. 3-MA + UFAs group. ^++^*p* < 0.01 vs. 3-MA + UFAs group. ^+++^*p* < 0.001 vs. 3-M0041 + UFAs group. ^$^*p* < 0.05 vs. TLR7KO + UFAs group. ^$$^*p* < 0.01 vs. TLR7KO + UFAs group. ^$$$^*p* < 0.001 vs. TLR7KO + UFAs group.

As confirmed above, TLR7 regulates IGF-1 balance between hepatocytes and the extracellular fluid via autophagy activation. We examined TLR7’s effect on lipid accumulation in hepatocytes to investigate the role of TLR7 in the “first hit”. The classic “first hit” is simple lipid accumulation in the liver. We hypothesized that TLR7 regulation of IGF-1 balance serves as a primary barrier against lipid accumulation. As shown in Fig. 3A, UFAs induced significant lipid accumulation, which stains lipid droplets red compared with non-treated controls. Lipid accumulation was slightly increased under 3-MA and TLR7KO conditions. Moreover, 3-MA or TLR7KO incubated with UFAs revealed massive lipid accumulation compared with each singly treated group. Imiquimod significantly reduced lipid accumulation due to UFAs, which was abrogated by 3-MA. Rapamycin also reduced lipid accumulation in TLR7KO. We examined the levels of adipogenic genes in corresponding samples through real-time RT-PCR (Fig. S1). Adipogenic genes showed similar trends in lipid accumulation results. Thus, TLR7 efficiently blockades lipid accumulation in hepatocytes. We examined the gene levels of IGF-1 before examining protein expression (Fig. 3B). IGF-1 gene levels did not differ significantly between the experimental samples. This suggests that IGF-1 synthesis is not affected by any other conditions. A previous study reported that liver IGF-I mRNA levels showed a negative relationship with the fibrosis score^29^. However, protein expression of IGF-1 showed significant differences in cell lysates. As shown in Fig. 3C, UFAs reduced the expression of LC3A/B while increasing the expression of IGF-1. 3-MA or TLR7KO incubated with UFAs abrogated the expression of LC3A/B while strongly inducing IGF-1 expression, compared with each singly-treated group. Imiquimod reversed LC3A/B reduction and IGF-1 increases in UFAs-treated cells, but 3-MA abrogated this imiquimod effect. Secreted IGF-1 was negatively correlated with protein expression in cell lysates (Fig. 3D). These results coincided with our previous hypothesis, which suggests regulation of IGF-1 balance between intracellular and extracellular fluid. Next, we investigated whether lipid accumulation induced by UFAs affected oxidative stress. As shown in Fig. 3E, ROS was increased during UFAs treatment. The graphic tendencies of ROS coincided with the histogram of IGF-1 expression in cell lysates (Fig. 3C,E). IGF-1 may potentiate ROS production in cell levels^30^. Moreover, different sources of IGF-1 induce the synthesis of lipid peroxidation products such as MDA and 4-HNE via oxidative stress^31^,^32^,^33^. Based on previous studies, intracellular IGF-1 served as a pathogenic factor that promotes NAFLD progression through oxidative stress induction. UFAs, which are comprised of arachidonic acid and oleic acid, are a precursor to lipid peroxidation. Cellular contents of MDA and 4-HNE were examined with corresponding samples (Fig. 3F,G). Naturally, UFAs significantly induced MDA and 4-HNE in hepatocytes, while imiquimod reversed the effect of UFAs. 3-MA and TLR7KO cells during UFAs treatment produced 3-fold increase in MDA and 4-HNE compared with each singly treated group. 3-MA abrogated the effect of imiquimod, which attenuated MDA and 4-HNE during UFAs treatment. Taken together, these results suggest that TLR7 serves as a barrier against lipid accumulation by regulating IGF-1 balance via autophagy activation. Thus, the cellular content of IGF-1 induced by massive lipid accumulation is linked to development of the “second hit,” which consists of lipid peroxidation products. To verify the effect of IGF-1 on MDA and 4-HNE, we examined protein expression of IGF-1, MDA, and 4-HNE using by Tet-On cells with or without IGF-1 siRNA (Fig. S2). Intrinsic induced IGF-1 produced MDA and 4-HNE, while intrinsic inhibited IGF-1 did not. Moreover, we examined whether treatment with IGF-1 recombinant protein counteracted the effect of imiquimod, which reduced protein levels of IGF-1, MDA, and 4-HNE (Fig. S3). IGF-1 treatment in TLR7KO cells strongly induced protein levels of IGF-1, MDA, and 4-HNE compared with the TLR7KO only group. It is quite evident that IGF-1 induced lipid peroxidation, which an etiological cause of the “second hit”.

### The lipid peroxidation products MDA and 4-HNE trigger massive NAFLD via suppression of TLR7: “Second hit” hypothesis confirmation

**Figure 4:**
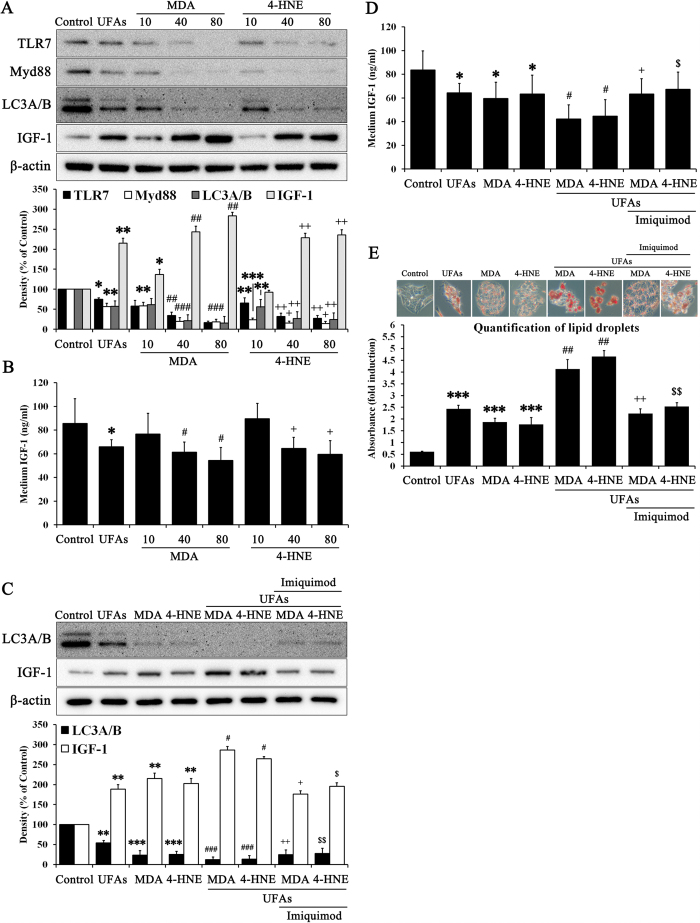
MDA and 4-HNE accelerate massive lipid accumulation by suppressing TLR7. Hepatocytes were treated with or without 10, 40, and 80 μM MDA and 4-HNE for 72 h. Immunoblotting was performed to evaluate (**A**) the protein expression of TLR7, Myd88, LC3A/B, IGF-1, and β-actin. (**B**) Medium from the cell supernatant was used to evaluate IGF-1 contents on ELISA. Data are mean and SEM values (*n* = 3). *p < 0.05 vs. untreated control. **p < 0.01 vs. untreated control. ***p < 0.001 vs. untreated control. ^#^*p* < 0.05 vs. MDA 10 group. ^##^*p* < 0.01 vs. MDA 10 group. ^###^*p* < 0.001 vs. MDA 10 group. ^+^*p* < 0.05 vs. 4-HNE 10 group. ^++^*p* < 0.01 vs. 4-HNE 10 group. Hepatocytes were treated with or without 40 μM MDA, 40 μM 4-HNE, and 10 μg/ml imiquimod in the presence or absence of 10 mM UFAs for 72 h. (**C**) We then performed immunoblotting to evaluate the protein expression of LC3A/B and IGF-1. (**D**) Medium from the cell supernatant was used to evaluate IGF-1 content on ELISA. (**E**) Oil Red O staining was performed to evaluate lipid accumulation. The red stained area indicated lipid droplets, which were quantified by absorbance measurement. Data are mean and SEM values (*n* = 3). *p < 0.05 vs. untreated control. **p < 0.01 vs. untreated control. ***p < 0.001 vs. untreated control. ^#^*p* < 0.05 vs. UFAs group. ^##^*p* < 0.01 vs. UFAs group. ^###^*p* < 0.001 vs. UFAs group. ^+^*p* < 0.05 vs. MDA + UFAs group. ^++^*p* < 0.01 vs. MDA + UFAs group. ^$^*p* < 0.05 vs. 4-HNE + UFAs group. ^$$^*p* < 0.01 vs. 4-HNE + UFAs group.

To identify how MDA and 4-HNE, which can cause the “second-hit” that leads to progression to severe NAFLD, we confirmed a putative pathway related to TLR7, which provides a protection against progressive NAFLD. A few studies reported an association between lipid peroxidation products and TLRs^34^,^35^,^36^. However, no studies have connected TLR7 and lipid peroxidation products. Treatment with MDA and 4-HNE attenuated protein expression of TLR7, Myd88, and LC3A/B, while increasing IGF-1 in a dose dependent manner (Fig. 4A). Unlike intracellular IGF-1, secreted IGF-1 decreased in a dose dependent manner, which was consistent with our previous data (Fig. 4B). Based on these results, we used adequate concentrations of MDA and 4-HNE (40 μM) for subsequent experiments. Each MDA and 4-HNE treatment reduced LC3A/B and increased IGF-1 during incubation with UFAs (Fig. 4C). Naturally, secreted IGF-1 was inversely correlated with intracellular IGF-1 (Fig. 4D). Imiquimod counteracted the deleterious effects of MDA and 4-HNE. As shown in Fig. 4E, MDA and 4-HNE exacerbated lipid accumulation alone and in UFAs treated cells. Lipid droplets were reduced by imiquimod in MDA and 4-HNE treated cells incubated with UFAs. We examined the levels of adipogenic genes through real-time RT-PCR (Fig. S4). Gene expression showed similar trends to lipid accumulation results. Taken together, MDA and 4-HNE produced by lipid accumulation with IGF-1 may exert second hit on unstable TLR7, and consequently decrease protections against NAFLD. These results suggest a pathogenic pathway leading to the “second hit” for feedback systems in NAFLD.

### NAFLD deteriorated severely in TLR7 knockout mice fed a UFAs diet: “First hit” hypothesis confirmation

**Figure 5:**
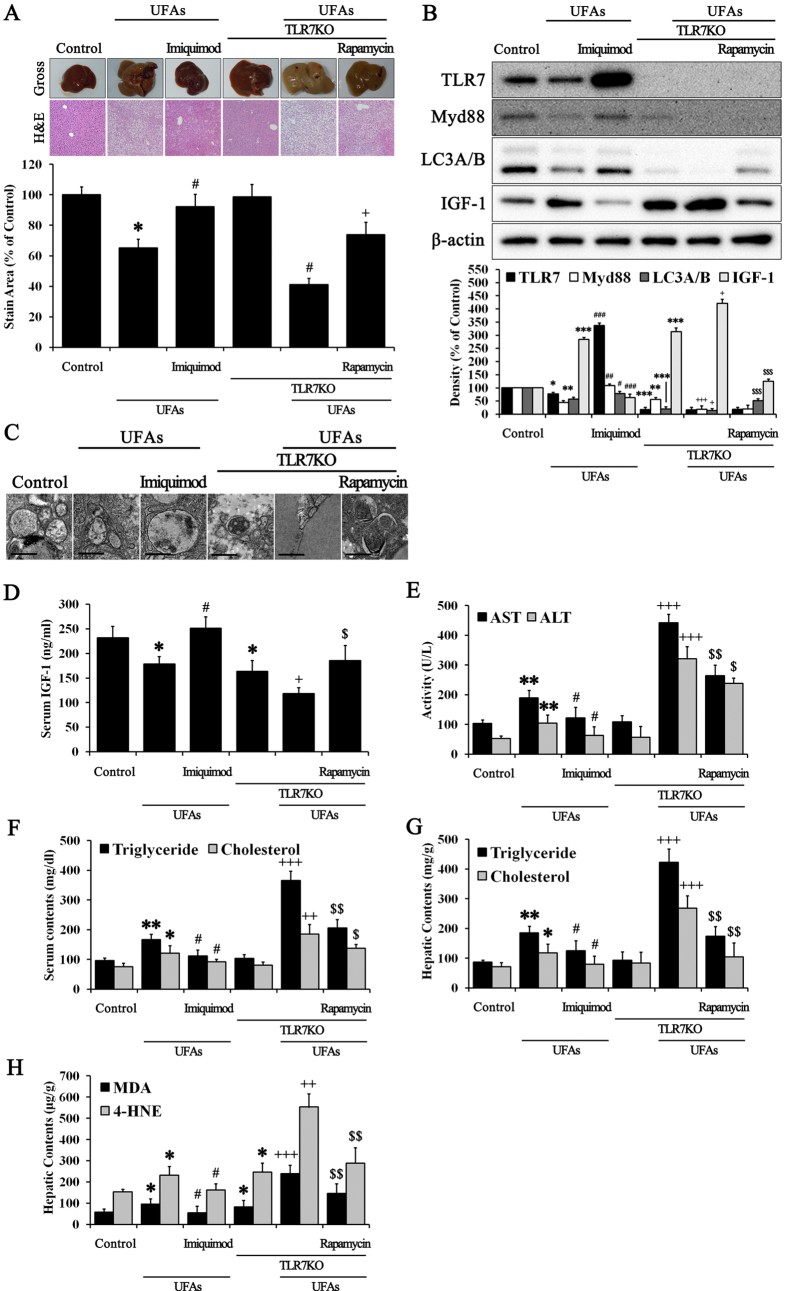
NAFLD was improved by TLR7-mediated regulation of IGF-1. Normal control mice and TLR7KO mice were injected with or without 0.1 mg/kg imiquimod and 2 mg/kg rapamycin twice a week while eating either a UFAs diet or a normal research diet for 8 weeks. (**A**) Histopathology analyses were performed, and we photographed the gross appearance of the liver and tissue section images stained with H & E (Indicated magnification: X200). The stained area was quantified with the Image Pro analysis program. Immunoblotting was performed to evaluate (**B**) the protein expression of TLR7, Myd88, LC3A/B, IGF-1, and β-actin in liver tissues. (**C**) Images of autophagosome formation were obtained from TEM in corresponding groups. The scale bar indicates 500 nm. Serum contents of (**D**) IGF-1, (**E**) AST, ALT, (**F**) triglycerides, and cholesterol were examined. Hepatic contents of (**G**) triglycerides, cholesterol, (**H**) MDA, and 4-HNE were examined. Data are mean and SEM values (*n* = 3). *p < 0.05 vs. untreated control mice. **p < 0.01 vs. untreated control mice. ***p < 0.001 vs. untreated control mice. ^#^*p* < 0.05 vs. control + UFAs group. ^##^*p* < 0.01 vs. control + UFAs group. ^###^*p* < 0.001 vs. control + UFAs group. ^+^*p* < 0.05 vs. TLR7 group. ^++^*p* < 0.01 vs. TLR7 group. ^+++^*p* < 0.001 vs. TLR7 group. ^$^*p* < 0.05 vs. TLR7KO + UFAs group. ^$$^*p* < 0.01 vs. TLR7KO + UFAs group. ^$$$^*p* < 0.001 vs. TLR7KO + UFAs group.

To evaluate the role of TLR7 in preventing NAFLD, we used TLR7KO mice fed a UFAs diet. As shown in Fig. 5A, gross and histopathologic observations in normal mice fed UFAs revealed stable NAFLD. Imiquimod treatment during the experimental period in UFAs-fed normal mice revealed improvement of NAFLD. Deleterious morphological changes were observed in the gross appearances of the livers from UFAs-fed TLR7KO mice. Compared with the normal mice fed UFAs, the gross liver specimens of TLR7KO mice fed UFAs changed significantly and were paler. These notable abnormalities are suggestive of those found in severe NAFLD, and were improved by rapamycin treatment. Histological analysis using H & E staining also revealed significant differences among all experimental groups. The liver sections of UFAs-fed TLR7KO mice exhibited severe ballooning degeneration. Rapamycin treatment in UFAs-fed TLR7KO mice decreased the size of the balloons and reduced their numbers. Numerical data of liver sections was digitized from taken images, and this data suggested that TLR7 prevented progressive NAFLD during UFAs induction. Any change that was not observed in only TLR7KO mice might be ascribable to the regulation of other preventive factors in the liver. UFAs-fed normal mice revealed stain area about 63% compared to control. In the TLR7KO mice, UFAs feeding leads to significant reduction of stain area about 60% compared to TLR7KO mice. These reductions of stain area were reversed by each imiquimod and rapamycin. Protein expression of TLR7, Myd88, LC3A/B, and IGF-1 were examined using tissue lysates from each experimental liver (Fig. 5B). Consistent with *in vitro* results, livers from UFAs-fed normal mice revealed lower expression of TLR7, Myd88, and LC3A/B and higher IGF-1 compared with normal mice fed a normal diet, while imiquimod treatment significantly counteracted the deleterious effects of UFAs. The expression of TLR7, Myd88, and LC3A/B vanished in UFAs-fed TLR7KO mice, while expression of IGF-1 increased notably. Rapamycin treatment in UFAs-fed TLR7KO mice increased expression of LC3A/B, but not TLR7 and Myd88. Accordingly, we observed autophagosome formation in all the experimental samples (Fig. 5C). TEM images revealed autophagosomes of various sizes and morphologies in liver tissues. Naturally, imiquimod and rapamycin induced the formation of large, round autophagosomes. The autophagosomes of UFAs-fed normal mice and TLR7KO mice were small with an indeterminate form. Subsequently, serum biochemistry was also performed. Serum levels of IGF-1 were consistent with *in vitro* results (Fig. 5D). Serum IGF-1 was reduced in UFAs-fed normal mice and TLR7KO mice. UFAs-fed TLR7KO mice revealed the lowest serum contents of IGF-1. Imiquimod and rapamycin counteracted the reduction in secreted IGF-1 that was induced by UFAs and UFAs with TLR7KO mice. These *in vivo* results support the idea that autophagy induced by TLR7 regulates transport of IGF-1 in and out of the liver. Other serum markers of liver conditions, such as AST, ALT, triglycerides, and cholesterol were examined, and exhibit similar results that are consistent with our hypothesis (Fig. 5E,F). Moreover, hepatic content of triglycerides, cholesterol, MDA, and 4-HNE were examined (Fig. 5G,H). These markers of liver condition were consistent with protein expression of IGF-1 in liver tissues. In addition, we quantified the levels of adipogenic genes in liver tissues through real-time RT-PCR (Fig. S5) and these were similar to results from progressive NAFLD. To investigate hepatic IGF-1 content in all stages of chronic liver disease, we performed comparative analysis of protein expression of TLR7, LC3A/B, and IGF-1 in livers from mice under normal, NAFLD, and cirrhosis conditions (Fig. S8). TLR7 and LC3A/B expression were reduced in both NAFLD and cirrhosis. However, IGF-1 expression was increased in NAFLD, but not cirrhosis. Accordingly, serum contents of IGF-1 decreased in both NAFLD and cirrhosis (Fig. S9). We thought that cirrhotic livers had the lowest IGF-1 levels caused by IGF-1 was exhausted and simply cannot produce IGF-1 under cirrhosis conditions. Taken together, these *in vivo* results suggested that TLR7 primarily prevents lipid accumulation and attenuates progressive NAFLD by regulating IGF-1 balance.

### Progression of NAFLD was massively and continuously accelerated by the lipid peroxidation products MDA and 4-HNE: “Second hit” hypothesis confirmation

**Figure 6:**
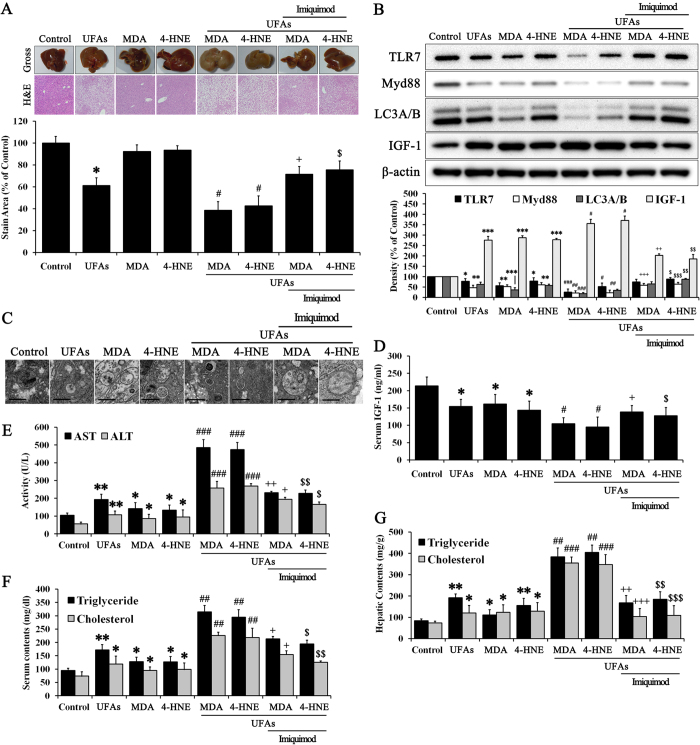
MDA and 4-HNE exacerbated progression of NAFLD via blockade of TLR7. Normal mice were injected with or without 0.4 mg/kg MDA, 0.8 mg/kg 4-HNE, and 0.1 mg/kg imiquimod twice a week while eating either a UFAs diet or a normal research diet for 8 weeks. (**A**) Histopathology analyses were performed and we photographed the gross appearance of the liver and tissue section images stained with H & E (Indicated magnification: X200). The stained area was quantified with the Image Pro analysis program. Immunoblotting was performed to evaluate (**B**) the protein expression of TLR7, Myd88, LC3A/B, IGF-1, and β-actin in liver tissues. (**C**) Images of autophagosome formation were obtained from TEM. The scale bar indicates 500 nm. We examined the serum contents of (**D**) IGF-1, (**E**) AST, ALT, (**F**) triglycerides, and cholesterol and the hepatic content of (**G**) triglycerides and cholesterol. Data are mean and SEM values (*n* = 3). *p < 0.05 vs. untreated control mice. **p < 0.01 vs. untreated control mice. ***p < 0.001 vs. untreated control mice. ^#^*p* < 0.05 vs. UFAs group. ^##^*p* < 0.01 vs. UFAs group. ^###^*p* < 0.001 vs. UFAs group. ^+^*p* < 0.05 vs. MDA + UFAs group. ^++^*p* < 0.01 vs. MDA + UFAs group. ^+++^*p* < 0.001 vs. MDA + UFAs group. ^$^*p* < 0.05 vs. 4-HNE + UFAs group. ^$$^*p* < 0.01 vs. 4-HNE + UFAs group. ^$$$^*p* < 0.001 vs. 4-HNE + UFAs group.

**Figure 7:**
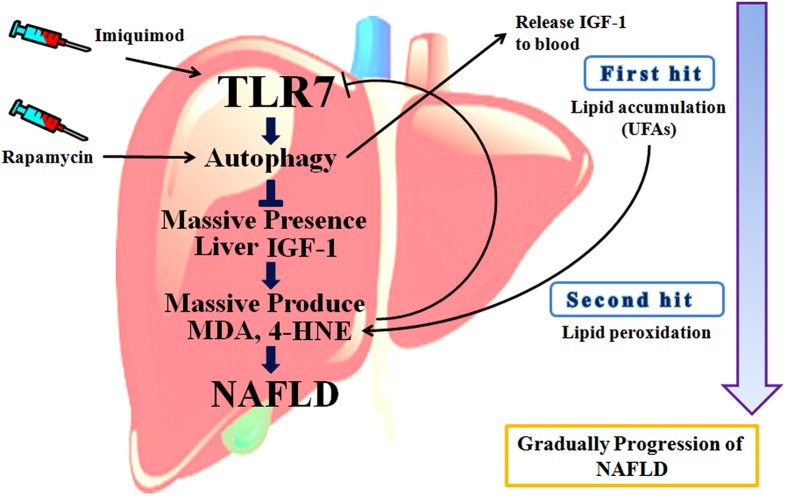
A schematic diagram of “two hit” hypothesis associated with TLR7, autophagy, IGF-1 and lipid peroxidation. The “two hit” hypothesis explains gradually progression of NAFLD. Lipid accumulation, indicate as “first hit” produces lipid peroxidation products such as MDA, and 4-HNE indicate as “second hit” gradually. The “second hit” reduced TLR7 and autophagy serially. This reduction of autophagy inhibits IGF-1 release from liver to blood and accumulates massive IGF-1 in liver. Massive presence of IGF-1 in liver induced massive MDA, and 4-HNE for progression of NAFLD. TLR7-Autophagy signaling usually protects against “first hit” and “second hit” via regulation of IGF-1 contents in the liver. TLR7 agonist, imiquimod and autophagy activator, rapamycin reversed previous phenomenon. In conclusion, TLR7 prevents progression of NAFLD, and serves a pivotal role in both the first and the second hits that lead to NAFLD.

Based on our *in vitro* results, in which IGF-1 induced lipid peroxidation products, we performed an *in vivo* experiment to identify whether IGF-1 injection changes the liver content of MDA and 4-HNE (Fig. S6). IGF-1 injection into normal mice induced MDA and 4-HNE production in liver tissues. This effect was accelerated when mice were fed UFAs. Thus, massive IGF-1 accumulation in the liver may abnormally produce MDA and 4-HNE. To demonstrate our hypothesis regarding the “second hit”, we injected MDA and 4-HNE into mice fed UFAs or a normal diet. As shown in Fig. 6A, injections of only MDA or 4-HNE induced a change in the gross appearance of the liver, causing pale and discolored margins. However, tissue sections of mice injected with only MDA or 4-HNE did not show notable change. The gross appearance of the liver in MDA or 4-HNE-injected UFAs-fed mice revealed deterioration; livers from these mice were enlarged and pale compared with the livers from mice injected with only MDA or 4-HNE. Massive ballooning and shrinking of the cellular matrix was also revealed in H & E stained liver sections from MDA or 4-HNE-injected UFAs-fed mice. Imiquimod improved the gross appearance and condition of tissue sections in MDA and 4-HNE-injected UFAs-fed mice. Subsequently, protein levels of TLR7, Myd88, LC3A/B, and IGF-1 were confirmed in liver (Fig. 6B). MDA and 4-HNE reduced the expression of TLR7, Myd88, and LC3A/B, while increasing IGF-1 compared with the untreated control. Added UFAs exacerbated these changes in protein expression. However, imiquimod reversed the reduction of TLR7, Myd88, and LC3A/B and the increase in IGF-1 in both MDA and 4-HNE-injected UFAs-fed mice. Autophagosome formation in both MDA and 4-HNE-injected UFAs-fed mice revealed an indeterminate form, while imiquimod reversed autophagosome formation (Fig. 6C). Serum biochemistry was performed, and these results were consistent with our *in vitro* results (Fig. 6D–F). MDA and 4-HNE reduced serum IGF-1 content, while this was exacerbated with UFAs (Fig. 6D). Imiquimod reversed the reduction of serum IGF-1 content in both MDA and 4-HNE-injected UFAs-fed mice. The levels of AST, ALT, triglycerides, and cholesterol in serum were also examined (Fig. 6E,F) and were consistent with our hypothesis and previous results. Hepatic triglyceride and cholesterol contents were similar to serum triglyceride and serum contents (Fig. 6G). Moreover, adipogenic gene expression, as quantified by qRT-PCR, was similar to trends in progressive NAFLD (Fig. S7). Taken together, these *in vivo* results suggest that both MDA and 4-HNE secondarily exacerbated NAFLD via feedback to inhibit TLR7. A summary of our findings is shown in Fig. 7.

## Discussion

TLRs play a pivotal role in innate immune responses against extrinsic pathogens such as microorganisms and it activates two independent pathways such as the MyD88 pathway for all TLRs except TLR3 and MyD88-independent for TLR3 and TLR4. These TLRs may also have a crucial role in the pathogenesis of chronic liver disease^13^,^14^,^37^,^38^. TLR4 and TLR2, which are major TLRs, have been best studied in NAFLD associated with inflammation^37^,^38^. These studies suggested that TLR4 can lead to progression of NAFLD, while TLR2 prevents NAFLD progression via uptake of diacylated lipoproteins. In human cases, TLR1-5 mRNAs are increased in the livers of NAFLD patients, but TLR6-10 mRNAs are not^12^. We investigated TLR7 as it most closely matched our hypothesis. TLRs including TLR7 initialize the signaling pathways that primary innate responses follow in the adaptive immune system^39^. Based on this crucial role in the induction of a strong anti-pathogen response, TLR7 was regarded as an attractive target for anti-viral therapy with TLR7 agonists^40^. These TLR7 agonists can be used in the liver to treat hepatitis C. TLR7 agonists such as resiquimod, isatoribine, and ANA975 ameliorated hepatitis C virus infection by inhibiting viral attachment and inducing the immune response^41^,^42^. During anti-viral treatment, TLR7 agonists exert anti-fibrotic effects in liver via their anti-angiogenic function, and a mechanistic role for TLR7 in signaling was found^14^,^43^. However, the role of TLR7 and the efficacy of its agonists in NAFLD, which ranges from simple steatosis that can progress to NASH and then to fibrosis or cirrhosis, is unknown. Moreover, there are many hypotheses regarding NAFLD progression, with little mechanistic validation. We investigated TLR7 and the “two-hit” hypothesis, which is a classical hypothesis that explains NAFLD progression.

We investigated two stages of NAFLD progression, to correspond with each of two hits. First, we explored the relationship between TLR7 and autophagy, as well as the role of TLR7 in IGF-1 secretion. We based this study on TLR7 as it is the first barrier against extrinsic stimulation and can therefore affect autophagy, which serves as a defense factor through the Myd88 signaling pathway^21^. Autophagy is the fundamental catabolic factor that degrades abnormal or unnecessary cellular components through lysosomal activity, thereby improving cellular survival^44^. During this process, targeted cytoplasmic constituents are within the autophagosome, which is a double-membraned vesicle. Under disease conditions, autophagy serves as an adaptive response to stress, which promotes survival in some cases and cell death and morbidity in others^45^. Recently a report on the pharmacological promotion of autophagy suggested a crucial role for autophagy in the improvement of steatosis in alcoholic and non-alcoholic fatty liver conditions^46^. Rapamycin, an autophagy inducer, enhanced degradation of lipids, or lipophagy, to improve steatosis. Thus, we confirmed an association between with TLR7 and autophagy in hepatocytes (Fig. 1). As expected, LC3A/B, which is a marker of autophagy activation, was regulated by TLR7 and Myd88 in hepatocytes. Autophagy activated by TLR7 reduced intracellular IGF-1 by increasing IGF-1 secretion (Fig. 2B,C). IGF-1 originates from hepatocytes in the liver and serves as an endocrine hormone, but can also drive target tissues in a paracrine or autocrine fashion^29^. Although the highest IGF-1 production occurs during puberty, it is produced throughout life. IGF-1 stimulates DNA synthesis, cell proliferation, amino acid uptake, and protein synthesis in a variety of tissues^47^. Low IGF-1 levels result in low protein synthesis in the senescent liver^48^, and the role of IGF-1 function in the liver has been investigated previously. IGF-1 deficiency improved hepatic function by activating autophagy^9^. Many studies have reported an association between IGF-1 and NAFLD and indicated that low circulating IGF-1 in serum may affect NAFLD pathogenesis^26^,^27^,^28^,^49^,^50^. Moreover, IGF-1 may accelerate adipogenesis^23^. We have not observed low IGF-1 in the livers of mice with NAFLD, though serum IGF-1 was low. Similarly, liver IGF-1 increases while serum IGF-1 decreases in diabetic mice^27^. We were skeptical about this contradiction between liver and serum IGF-1 content and therefore tested this in our model. IGF-1 was not reduced and did not disappear in liver tissues or serum in our results. It seems to be secreted into from cells into the external fluid. In fact, the classical secretion pathway involves GH stimulation of IGF-1 secretion from hepatocytes^51^. We hypothesize that activated autophagy secretes IGF-1 to the external fluid, possibly through its biomolecule transport function^22^. To support this result, we used engineered IGF-1-GFP induced cells (Fig. 2D–F).

Based on these results, we verified TLR7 as a primary barrier against UFAs-induced lipid accumulation (Fig. 3). Our results suggested that the presence of TLR7 or TLR7 agonists notably prevents lipid accumulation, which serves as the “first hit” in NAFLD. Moreover, the change in IGF-1 balance between the intracellular and extracellular fluid was consistent with our hypothesis, which involved IGF-1 release from cells due to autophagy activation (Fig. 3C,D). *In vivo* experiments confirmed these results (Fig. 5). UFAs-induced lipid accumulation in the liver was prevented by TLR7 or imiquimod, a TLR7 agonist. Liver contents of IGF-1 were released to serum by autophagy activation. These results suggest that lipid accumulation, which usually serves as the “first hit,” was usually prevented by TLR7, which produces an initial immune barrier by regulating IGF-1 presence in cells. However, the accumulation of continuous cellular damage induced lipid peroxidation products such as MDA and 4-HNE (Figs 3F,G and 5H). Moreover, a different source of IGF-1 induced synthesis of lipid peroxidation products via oxidative stress^31^,^32^,^33^. MDA is regarded as the most mutagenic lipid peroxidation product, while 4-HNE is the most toxic^52^. MDA is used as a biomarker for lipid peroxidation of omega-3 and omega-6 fatty acids^53^. 4-HNE is a cytotoxic product originating from the peroxidation of liver microsomal lipids^54^. These residues of lipid peroxidation products exert various harmful effects in the body^18^,^32^,^36^. However, few studies about the function of these lipid peroxidation products have been reported. We drew inspiration from one of study about lipid peroxidation products, which suggested that 4-HNE may reduce TLR4 responses^36^. TLRs initialize the innate immune response to protect against extrinsic pathogens, and we hypothesized that MDA and 4-HNE may disturb these primary immune barriers, such as lipid accumulation in liver. Thus, the second stage of our study elucidated whether MDA and 4-HNE could exert deleterious effects on TLR7 levels and clarified the possible mechanisms underlying the role of TLR7 in preventing progressive NAFLD. As shown in Fig. 4, MDA and 4-HNE significantly reduced TLR7 and its downstream signaling pathway to LC3A/B. The change in IGF-1 contents between the intracellular and extracellular fluid was consistent with previous results (Fig. 4A,B). MDA or 4-HNE incubated with UFAs induced massive lipid accumulation that was reversed by imiquimod (Fig. 4E). These *in vitro* results were reprised in *in vivo* experiments. As shown in Fig. 6, MDA and 4-HNE accelerate the progression of NAFLD via blockade of the TLR7 signaling pathway. The association between liver and serum IGF-1 content was also affected by MDA or 4-HNE injection (Fig. 6B,D). Our results demonstrated that TLR7 is regarded as crucial barrier against progressive NAFLD, and clarified the role of autophagy, IGF-1, and lipid peroxidation products such as MDA and 4-HNE, which are associated with TLR7 at the cellular and tissue levels in NAFLD. To expand these results, we performed comparative analysis of TLR7, LC3A/B, and IGF-1 in mice under normal, NAFLD, and cirrhosis conditions (Figs S8 and S9). NAFLD is a progressive form of chronic liver disease while cirrhosis is the end form of chronic liver disease, and so a cirrhotic liver is regarded as senescent liver and does not produce and maintain intrinsic IGF-1, as its capacity has been exhausted^48^.

In conclusion, the present study shows reasonable mechanisms involved in the “two-hit” hypothesis. TLR7 usually counteracts lipid accumulation, serves as the “first hit” by regulating IGF-1 content in the liver. MDA and 4-HNE serve as the “second hit” and were massively synthesized by impairment of lipid degeneration due to IGF-1. MDA and 4-HNE serve as the second hit to disrupt TLR7, which appears to be involved in a feedback system. This functional relationship is described in Fig. 7. Based on these findings, we suggest that TLR7 could serve as a novel therapeutic target for the prevention and treatment of NAFLD through improved immune responses, and that TLR7 may be involved in the “two-hit” hypothesis. This conclusion warrants further investigation regarding the role of TLR7 in clinical trials.

## Additional Information

**How to cite this article**: Kim, S. *et al*. Toll-like receptor 7 affects the pathogenesis of non-alcoholic fatty liver disease. *Sci. Rep.* **6**, 27849; doi: 10.1038/srep27849 (2016).

## Supplementary Material

Supplementary Information
