# Supplementary material for: Toll-like receptor 7 affects the pathogenesis of non-alcoholic fatty liver disease
Source: Sci Rep. 2016 Jun 9;6:27849. doi: 10.1038/srep27849 (PMC4899790; doi:10.1038/srep27849)
Supplement: Supplementary Information [file srep27849-s1.doc]

**Running title: TLR7 has a role in the “two-hit hypothesis”**

**Title: Toll-like receptor 7 affects the pathogenesis of non-alcoholic fatty liver disease**

Sokho Kim^1^, Surim Park^2^, Bumseok Kim^2^, and Jungkee Kwon^1*^

^1^Department of Laboratory Animal Medicine, ^2^Laboratory of Pathology, College of Veterinary Medicine, Chonbuk National University, 79 Gobongro, Iksan, 54596, Republic of Korea.

**Corresponding author**

Jungkee Kwon*

Department of Laboratory Animal Medicine, College of Veterinary Medicine, Chonbuk National University, 79 Gobongro, Iksan, 570-752, Republic of Korea. Tel: +82-63-270-3884; Fax: +82-63-270-3780; E-mail: jkwon@jbnu.ac.kr

**Supplementary information**

**Materials and Methods**

**Animals and *in vivo* experimental design**

We used 8-week old TLR7-deficient mice (TLR7KO) in these studies (25-30 g body weight). Dr. Akira (Osaka University, Suita, Japan) kindly provided the TLR7KO mice on a C57BL/6 background. Normal C57BL/6 mice were purchased from the SLC (Hamamatsu, Japan) and used as wild type control. The mice were maintained in microisolator cages under pathogen-free conditions on a 12-h/12-h light/dark cycle, and housed under controlled temperature (23±3°C, mean±range) and humidity (about 60%) conditions. All animals were cared for in accordance with institutional ethical guidelines for the care and use of experimental animals at Chonbuk National University. The animal facility of the Chonbuk National University is fully accredited by the National Association of Laboratory Animal Care. To establish NAFLD in experimental mice, UFAs was included in a modified mouse diet based on the normal research diet (Research Diets, NJ, USA), and contained 45% of its calories from fat. Detailed components of the experimental diet are described in Table S1. Each group consisted of 6 mice, and the normal control group was fed a normal control diet, while NAFLD induction groups were fed a UFAs diet for eight weeks. Cirrhosis induction groups were fed a UFAs diet for 40 weeks. During the experimental period, we administered IGF-1 (0.1 mg/kg) in 0.1 ml saline intraperitoneally twice a week to mice in specific experimental groups. At the end of the experimental period, food was withheld for 12 h and the mice were anesthetized with ether. Blood samples were taken from the inferior vena cava to analyze serum biomarkers. Subsequently, the livers were harvested and processed for immunoblotting; samples were stored at −80°C until analysis.

**Cell culture and treatment**

Hepatocytes were isolated from C57BL/6 and TLR7KO mice as previously described [20]. Cells were cultured at 37°C in a humidified, 5% CO_2_ atmosphere in Dulbecco’s Modified Eagle’s Medium with 10% fetal bovine serum (FBS), 100 U/ml penicillin, and 100 μg/ml streptomycin. After cell stabilization, hepatocytes were serum starved for 6 h and then treated with imiquimod (10 μg/ml), rapamycin (50 μM), 3-Methyladenine (3-MA; 10 mM), MDA (40 μM), 4-HNE (40 μM), IGF-1 (100 ng/ml), or IGF-1 siRNA, with or without scrambled siRNA, in the presence or absence of UFAs (10 mM) for 72 h. In addition, we purchased a HepG2 Tet-On® Advanced Cell Line transfected with IGF-1-GFP (Tet-On cells) from Clontech (CA, USA) and used it to examine the release of intrinsic inducible IGF-1. IGF-1-GFP induced by DOX (1 μg/ml) treatment for 72 h was incubated with a corresponding treatment such as IGF-1 siRNA, with or without scrambled siRNA, in the presence or absence of UFAs (10 mM).

**Small interfering RNA transfection**

Cells were plated in the indicated plastic ware until reaching confluence and were then starved for 6 h. The DharmaFECT 1 small interfering RNA (siRNA) Transfection Reagent (Dharmacon, Denver, CO, USA) was used to transfect the cells with 50 nM IGF-1 siRNA or scrambled siRNA oligonucleotides (Dharmacon) according to the manufacturer’s instructions and as reported previously.

**RNA preparation and real-time RT-PCR**

Total RNA was isolated from cells and precipitated with Ribo EX (Geneall, Daejeon, Korea) following the manufacturer’s protocols. The mRNA was reverse transcribed to cDNA using a Maxime RT PreMix kit (Intron, Seongnam, Korea) following the manufacturer’s protocols. For real-time RT-PCR, cDNA was amplified using a Mastercycler Gradient 5331 Thermal Cycler (Eppendorf, Germany). Real-time PCR runs were monitored by measuring the fluorescence signal after each cycle with an ABI Step One Plus Sequence Detection System (Applied Biosystems, Singapore). Specific primers for each gene were designed using Primer Express software (Applied Biosystems). The following forward and reverse primers were used for real-time RT-PCR quantification (forward and reverse): forward 5’-GCAGCCACCATCTAGCCTG-3’ and reverse 5’- CAGCAGTGAGTCTGCCTTGAT-3’ for sterol regulatory-element binding protein-1 (SREBP-1); forward 5’-CCTGGATAGCATTCCGAACCT-3’ and reverse 5’-AGCACATCTCGAAGGCTACACA-3’ for fatty acid synthase (FAS); forward 5’-CAAGAATACCAAAGTGCGATCAA -3’ and reverse 5’-GAGCTGGGTCTTTTCAGAATAATAAG-3’ for peroxisome proliferator-activated receptor γ (PPAR-γ); forward 5’-GCCAGACACCCCTGCTA-3’ and reverse 5’-GTTCTGGGCGTCACTCC-3’ for fatty acid binding protein 4 (aP2); and forward 5’-GCATGGCCTTCCGTGTTC-3’ and reverse 5’- GATGTCATCATACTTGGCAGGTTT-3’ for Glyceraldehyde-3-phosphate dehydrogenase (GAPDH), the housekeeping gene used as an internal control. All experiments were performed at least three times.

**Immunoblotting**

Total proteins from cell lysates and liver tissue lysates were subjected to sodium dodecyl sulfate polyacrylamide gel electrophoresis using 10% to 15% gels. Proteins were then electrophoretically transferred to polyvinylidene difluoride membranes (Bio-Rad Laboratories, Hercules, CA, USA). Membranes were blocked in 5% skim milk in PBS and then incubated with primary antibodies (IGF-1, MDA, 4-HNE, TLR7, LC3A/B, or β-actin), which were diluted 1:1,000 in 1% skim milk in PBS overnight at 4°C. Membranes were then incubated with peroxidase-conjugated anti-rabbit, anti-goat, or anti-mouse IgG antibodies (1:5,000; Millipore, Bedford, MA, USA) for 1 h. Immunoreactive bands were visualized with SuperSignal West Dura Extended Duration Substrate (Thermo Scientific, San Jose, CA, USA) and analyzed using a chemiImager analyzer system (Alpha Innotech, San Leandro, CA, USA).

**Enzyme-linked immunosorbent assay (ELISA)**

Serum content of IGF-1 (mean circulating whole-body) was measured by ELISA using a kit according to the manufacturer's instructions (Abcam, Cambridge, MA, USA). Absorbance at 560 nm was measured at using a microplate reader.

**Statistical analysis**

Results are presented as means ± standard error. Data were analyzed using the Student`s *t*-test (for two groups) or one-way ANOVA and Tukey’s test (for more than two groups). *P* values<0.05 were considered statistically significant. All analyses were performed using the Statistical Package for Social Sciences, version 13.0, for Windows (SPSS, Inc., Chicago, IL, USA).

**Results**

**Levels of adipogenic genes were examined in each corresponding result**

Several results for adipogenic genes, such as SREBP-1, FAS, PPAR-γ, and aP2, are examined in Fig. S1, Fig. S4, Fig. S5, and Fig. S7. These results were consistent with other NAFLD results. Adipogenic genes are regarded as markers of lipogenesis and lipid accumulation in hepatocytes and the liver. Thus, these results supported other experimental results in this manuscript.

**Engineered induction of intrinsic IGF-1 accelerates lipid peroxidation with or without additional UFAs**

To evaluate whether the natural presence of IGF-1 in hepatocytes affected lipid peroxidation, Tet-On cells were used for immunoblotting. As shown in Fig. S2, DOX induced intrinsic IGF-1, MDA, and 4-HNE expression, and accelerates their expression in the presence of UFAs. IGF-1 siRNA inhibited the expression of MDA and 4-HNE with or without UFAs. Thus, these results suggested that IGF-1 induces lipid peroxidation and accelerates the production of their products.

**Extrinsic IGF-1 treatment also induced lipid peroxidation**

To verify the effects of intrinsic IGF-1 on lipid peroxidation, we examined the effects of extrinsic IGF-1 on lipid peroxidation products. IGF-1 treatment accelerated its own expression, as well as MDA and 4-HNE (Fig. S3). IGF-1 treatment counteracted the effects of imiquimod on the expression of IGF-1, MDA, and 4-HNE. These results were consistent with effect of TLR7 on lipid peroxidation products. Moreover, we examined the effect of extrinsic IGF-1 in UFAs-fed or control mice (Fig. S6). Protein from liver tissues revealed interesting results. Only normal mice injected with IGF-1 showed increases in expression of IGF-1, MDA, and 4-HNE. Massive increases in IGF-1, MDA, and 4-HNE were revealed in IGF-1-injected UFAs-fed mice. These results suggest that massive IGF-1 accelerates the production of lipid peroxidation.

**Comparative analysis of TLR7, LC3A/B, and IGF-1 in each stage of chronic liver disease**

To clarify the role of IGF-1 content in each stage of chronic liver disease, we investigated IGF-1 levels in both liver and serum. As shown in Fig. S8, cirrhotic livers had the lowest protein expression of all the groups. TLR7 and LC3A/B expression gradually reduced from NAFLD to cirrhosis. Interestingly, IGF-1 expression in livers with NAFLD significantly increased compared with normal liver, while IGF-1 expression vanished in cirrhotic livers. Accordingly, we also examined secreted IGF-1 in each stage of chronic liver disease (Fig. S9). As shown in Fig. S9, secreted IGF-1 gradually reduced from NAFLD to cirrhosis. Moreover, comparative level of IGF-1 in serum from NAFLD was contrary to liver contents of IGF-1 in same group. These results may be caused by blockade in IGF-1 release from livers with NAFLD. We hypothesize that cirrhotic livers had the lowest IGF-1 levels because these are exhausted and simply cannot produce IGF-1 under those conditions.

**Table**

**Table S1. Detailed components of experimental diet**

| **Ingredient (g/kg)** | **Normal research diet** | **10% Mixed Fat** | **Ingredient(g/kg)** | **UFAs Diet** | **45% Mixed Fat** |
| --- | --- | --- | --- | --- | --- |
|  | **gram** | **Kcal** |  | **gram** | **Kcal** |
| **Casein** | **200** | **800** | **Casein** | **200** | **800** |
| **L-Cystine** | **3** | **12** | **L-Cystine** | **3** | **12** |
|  |  |  |  |  |  |
| **Corn Starch** | **315** | **1260** | **Corn Starch** | **72.8** | **291** |
| **Maltodextrin** | **35** | **140** | **Maltodextrin** | **100** | **400** |
| **Sucrose** | **350** | **1400** | **Sucrose** | **172.8** | **691** |
|  |  |  |  |  |  |
| **Cellulose** | **50** | **0** | **Cellulose** | **50** | **0** |
|  |  |  |  |  |  |
| **Soybean Oil** | **25** | **225** | **Oleic Acid** | **97.5** | **877.5** |
| **Lard** | **20** | **180** | **Arachidonic Acid** | **105** | **945** |
|  |  |  |  |  |  |
| **Mineral Mix** | **10** | **0** | **Mineral Mix** | **10** | **0** |
| **DiCalcium Phosphate** | **13** | **0** | **DiCalcium Phosphate** | **13** | **0** |
| **Calcium Carbonate** | **5.5** | **0** | **Calcium Carbonate** | **5.5** | **0** |
| **Potassium Citrate, 1 H2O** | **16.5** | **0** | **Potassium Citrate, 1 H2O** | **16.5** | **0** |
|  |  |  |  |  |  |
| **Vitamin Mix** | **10** | **40** | **Vitamin Mix** | **10** | **40** |
| **Choline Bitratrate** | **2** | **0** | **Choline Bitratrate** | **2** | **0** |
|  |  |  |  |  |  |
| **Total** | **1055** | **4057** |  | **858.1** | **4056.5** |
|  |  |  |  |  |  |
| **Nutrition rate(%)** | **g%** | **Kcal%** |  | **g%** | **Kcal%** |
|  |  |  |  |  |  |
| **Carbohydrate** | **67.3** | **70** | **Carbohydrate** | **41** | **35** |
| **Protein** | **19.2** | **20** | **Protein** | **24** | **20** |
| **Fat** | **4.3** | **10** | **Fat** | **24** | **45** |
|  |  |  |  |  |  |
| **Total Kcal/g** | **3.85** |  |  | **4.73** |  |

**Figures**

**
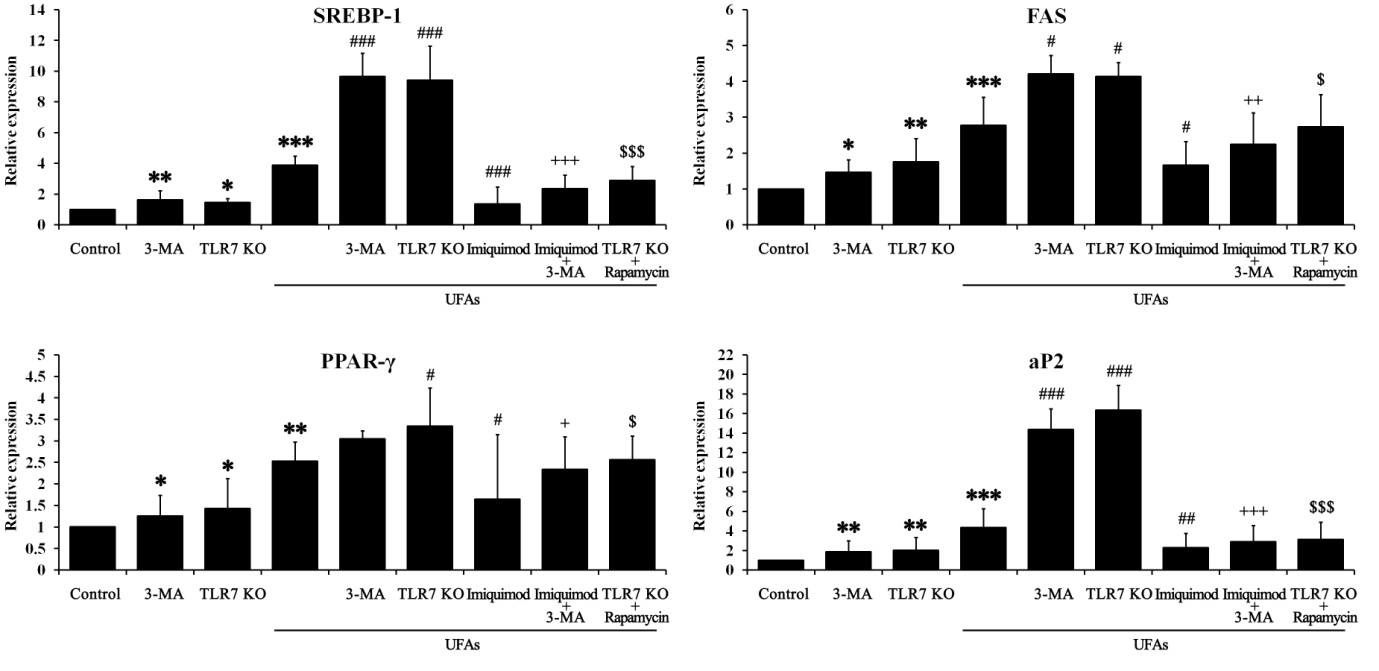
**

**Fig. S1. UFAs-induced lipid accumulation was prevented by TLR7, followed by autophagy activation**

Hepatocytes and TLR7KO hepatocytes were treated with or without 50 μM rapamycin, 10 mM 3-MA, and 10 μg/ml imiquimod in the presence or absence of 10 mM UFAs for 72 h. We examined the gene expression of SREBP-1, FAS, PPAR-γ, and aP2 in cells by real-time RT-PCR. Data are mean and SEM values (*n*=3). **p*<0.05 vs. untreated control. ***p*<0.01 vs. untreated control. ****p*<0.001 vs. untreated control. *^#^p*<0.05 vs. UFAs group. *^##^p*<0.01 vs. UFAs group. *^###^p*<0.001 vs. UFAs group. *^+^p*<0.05 vs. 3-MA + UFAs group. *^++^p*<0.01 vs. 3-MA + UFAs group. *^+++^p*<0.001 vs. 3-MA + UFAs group. *^$^p*<0.05 vs. TLR7KO + UFAs group. *^$$^p*<0.01 vs. TLR7KO + UFAs group. *^$$$^p*<0.001 vs. TLR7KO + UFAs group.

**
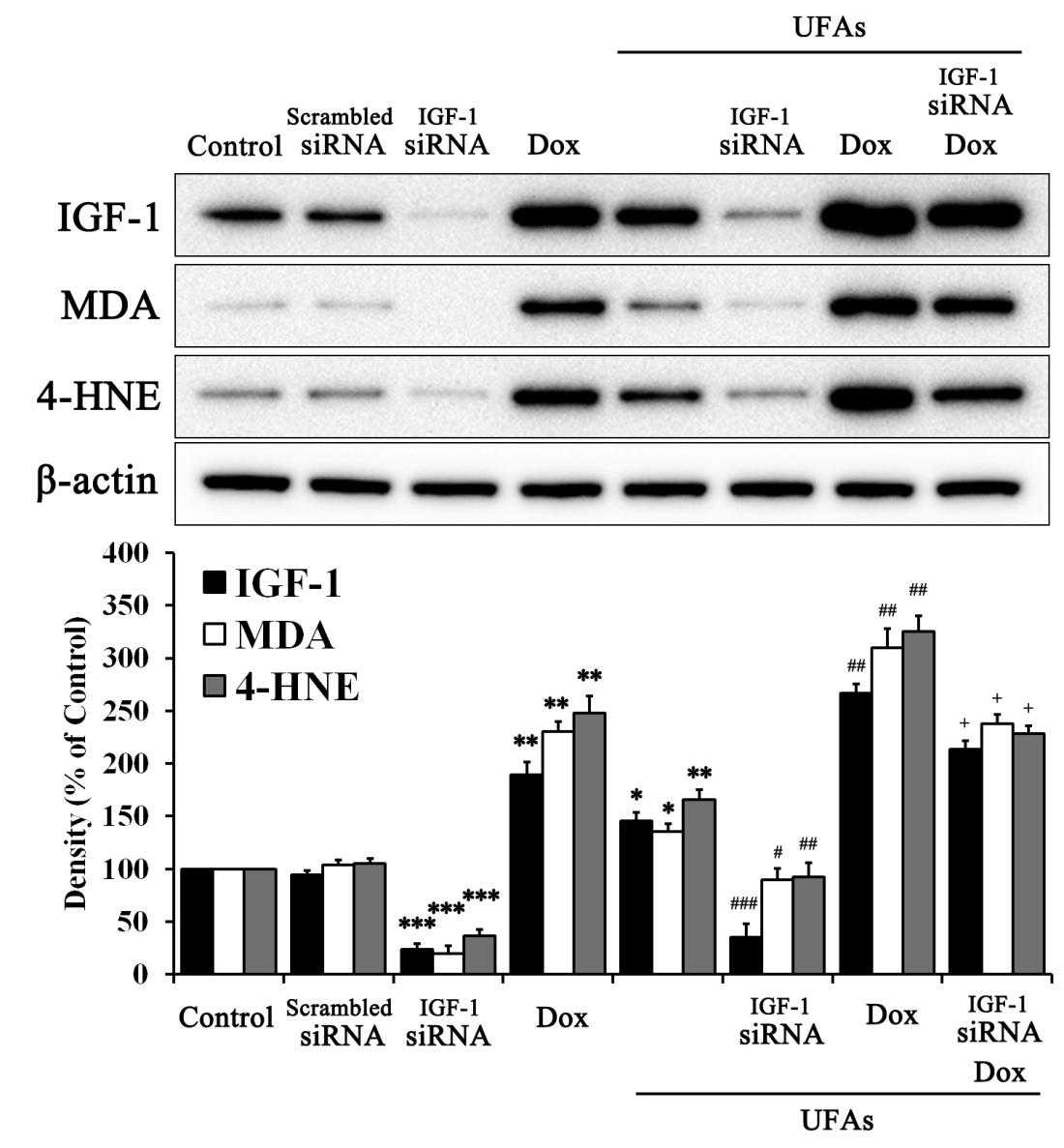
**

**Fig. S2. Intrinsic IGF-1 induced lipid peroxidation**

Tet-On cells were treated with or without 1 μg/ml DOX, IGF-1 siRNA, and scrambled siRNA in the presence or absence of 10 mM UFAs for 72 h. We performed immunoblotting to evaluate the protein expression of IGF-1, MDA, and 4-HNE. Data are mean and SEM values (*n*=3). **p*<0.05 vs. untreated control. ***p*<0.01 vs. untreated control. ****p*<0.001 vs. untreated control. *^#^p*<0.05 vs. UFAs group. *^##^p*<0.01 vs. UFAs group. *^###^p*<0.001 vs. UFAs group. *^+^p*<0.05 vs. DOX + UFAs group. *^++^p*<0.01 vs. DOX + UFAs group. *^+++^p*<0.001 vs. DOX + UFAs group.

**
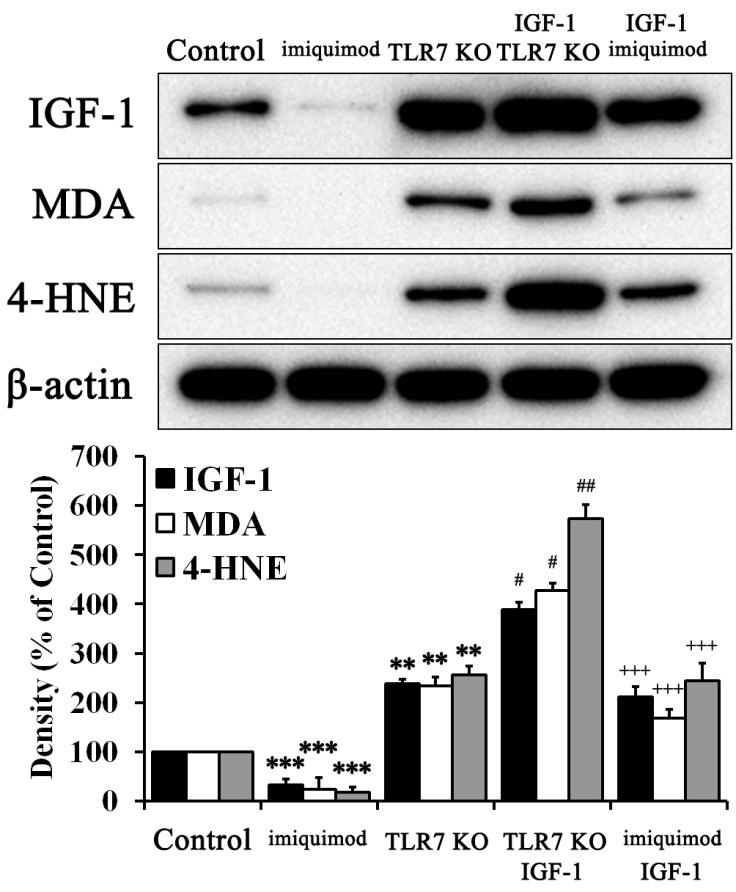
**

**Fig. S3. Extrinsic IGF-1 induced lipid peroxidation**

Hepatocytes and TLR7KO hepatocytes were treated with or without 10 μg/ml imiquimod and 100ng/ml IGF-1 for 72 h. We performed immunoblotting to evaluate the protein expression of IGF-1, MDA, and 4-HNE. Data are mean and SEM values (*n*=3). ***p*<0.01 vs. untreated control. ****p*<0.001 vs. untreated control. *^#^p*<0.05 vs. TLR7KO group. *^##^p*<0.01 vs. TLR7KO group. *^+++^p*<0.001 vs. imiquimod group.


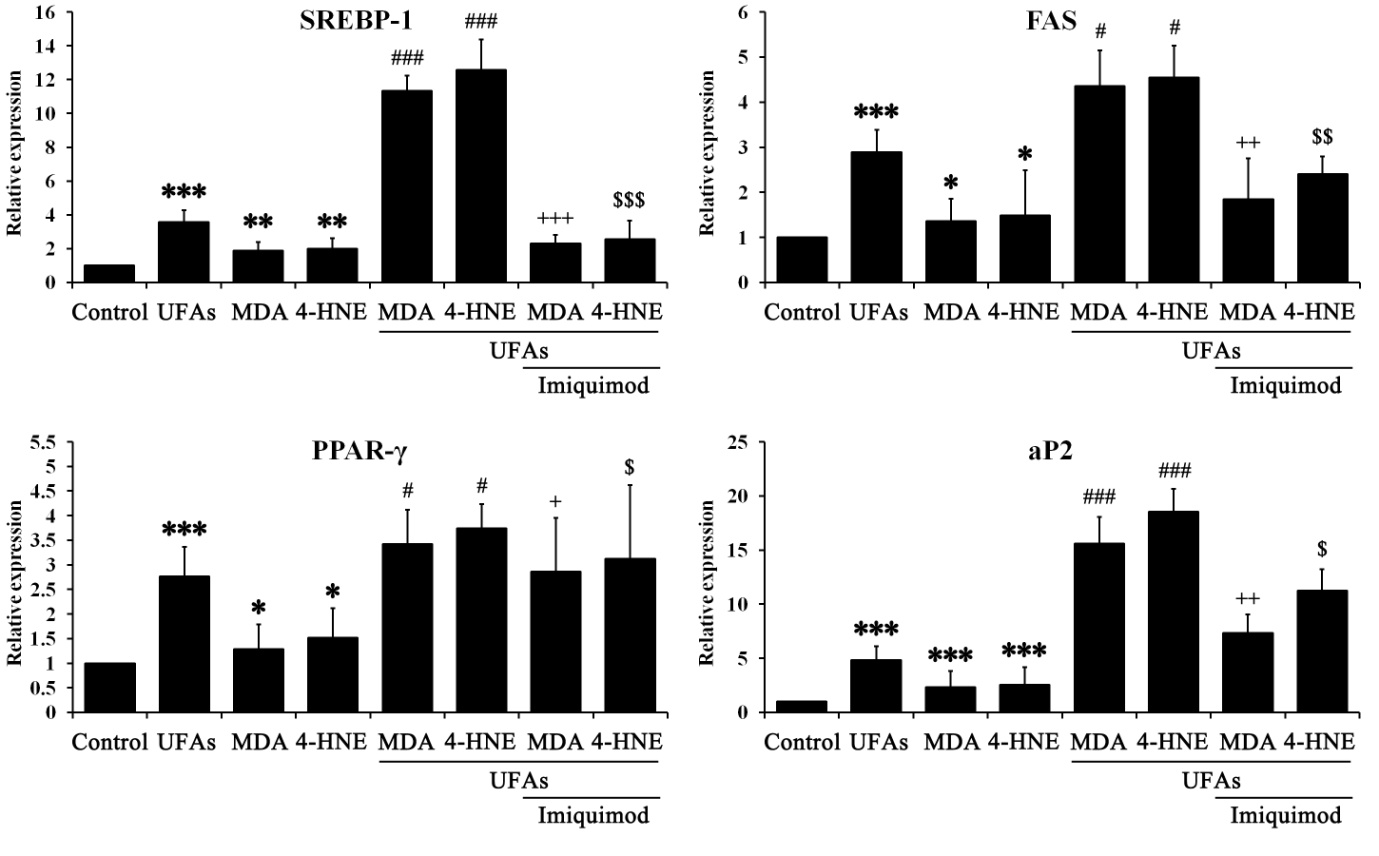


**Fig. S4. MDA and 4-HNE accelerate massive lipid accumulation by suppressing TLR7**

Hepatocytes were treated with or without 40 μM MDA, 40 μM 4-HNE, and 10 μg/ml imiquimod in the presence or absence of 10 mM UFAs for 72 h. Gene expression of SREBP-1, FAS, PPAR-γ, and aP2 in cells was examined by real-time RT-PCR. Data are mean and SEM values (*n*=3). **p*<0.05 vs. untreated control. ***p*<0.01 vs. untreated control. ****p*<0.001 vs. untreated control. *^#^p*<0.05 vs. UFAs group. *^##^p*<0.01 vs. UFAs group. *^###^p*<0.001 vs. UFAs group. *^+^p*<0.05 vs. MDA + UFAs group. *^++^p*<0.01 vs. MDA + UFAs group. *^$^p*<0.05 vs. 4-HNE + UFAs group. *^$$^p*<0.01 vs. 4-HNE + UFAs group.


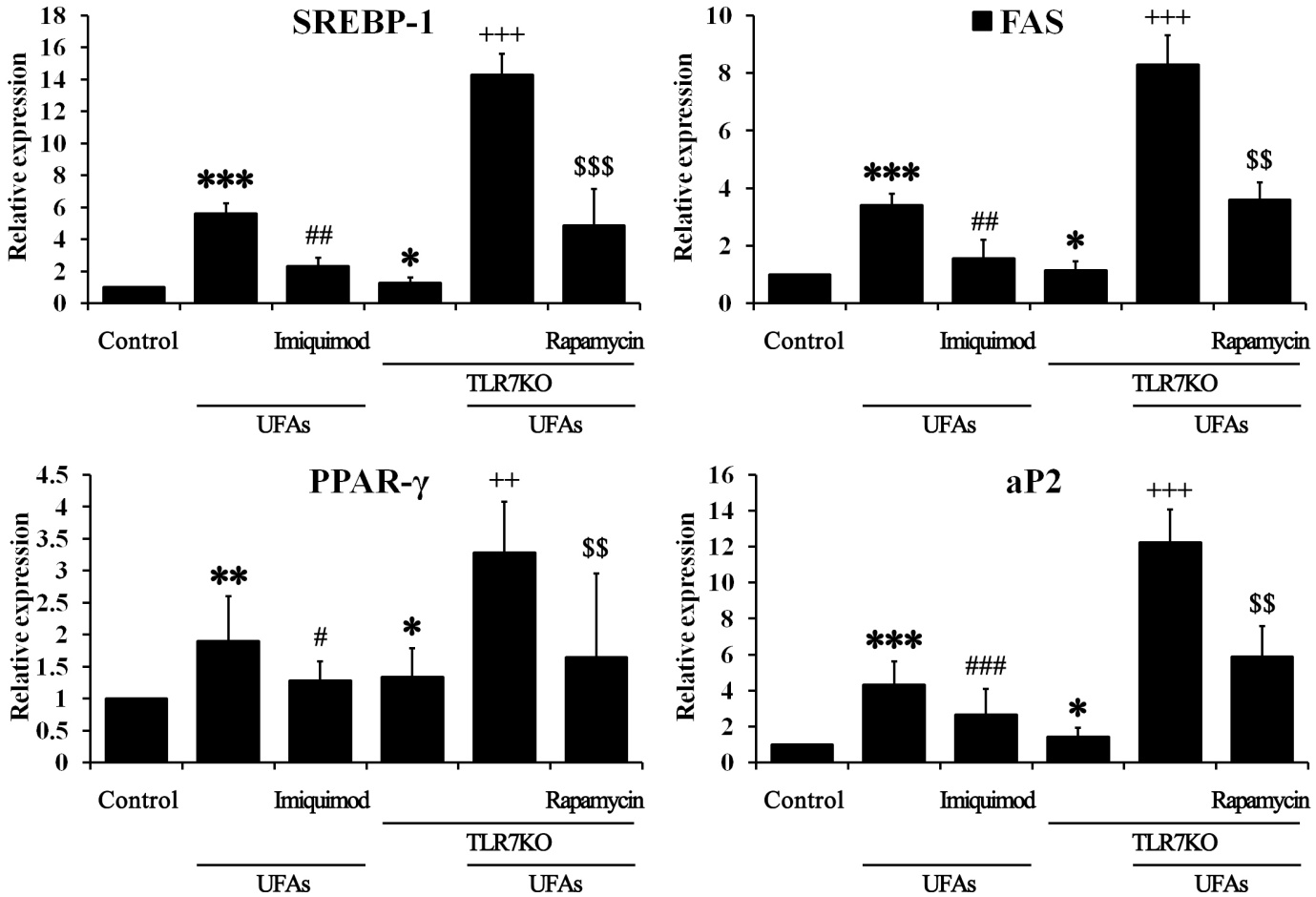


**Fig. S5. NAFLD was improved by TLR7 responses via IGF-1 regulation**

Normal control mice and TLR7KO mice were injected with or without 0.1 mg/kg imiquimod and 2 mg/kg rapamycin twice a week while eating a UFAs diet or a normal research diet for 8 weeks. Gene expression of SREBP-1, FAS, PPAR-γ, and aP2 in liver tissues was examined by real-time RT-PCR. Data are mean and SEM values (*n*=3). **p*<0.05 vs. untreated control mice. ***p*<0.01 vs. untreated control mice. ****p*<0.001 vs. untreated control mice. *^#^p*<0.05 vs. control + UFAs group. *^##^p*<0.01 vs. control + UFAs group. *^###^p*<0.001 vs. control + UFAs group. *^+^p*<0.05 vs. TLR7 group. *^++^p*<0.01 vs. TLR7 group. *^+++^p*<0.001 vs. TLR7 group. *^$^p*<0.05 vs. TLR7KO + UFAs group. *^$$^p*<0.01 vs. TLR7KO + UFAs group. *^$$$^p*<0.001 vs. TLR7KO + UFAs group.

**
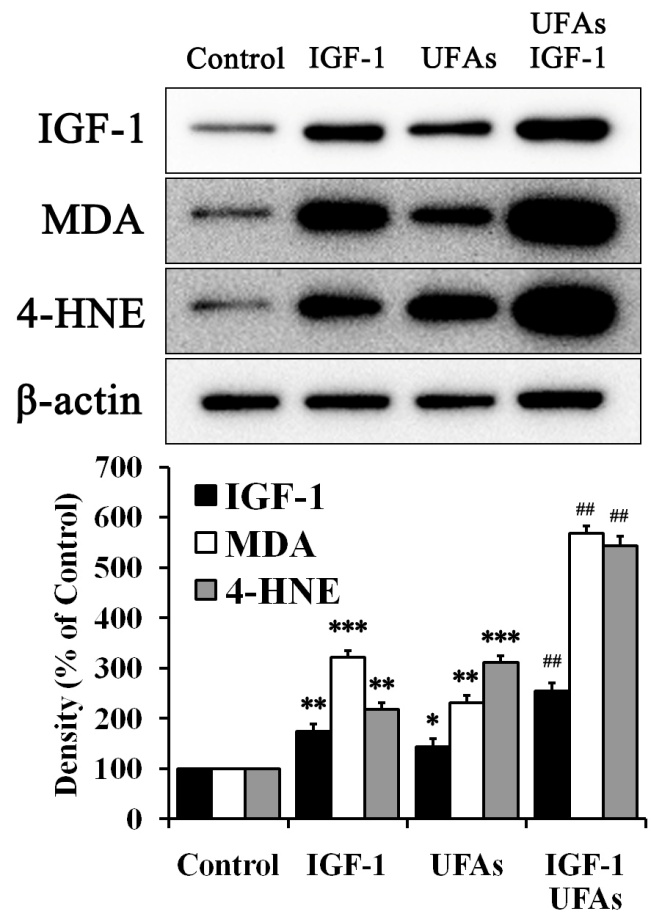
**

**Fig. S6. IGF-1 induced lipid peroxidation in UFAs fed mice**

Normal mice were injected with or without 0.1 mg/kg IGF-1 twice a week while eating a UFAs diet or a normal research diet for 8 weeks. Immunoblotting to evaluate the protein expression of IGF-1, MDA, and 4-HNE was performed. Data are mean and SEM values (*n*=3). **p*<0.05 vs. untreated control. ***p*<0.01 vs. untreated control. ****p*<0.001 vs. untreated control. *^##^p*<0.01 vs. UFAs group.


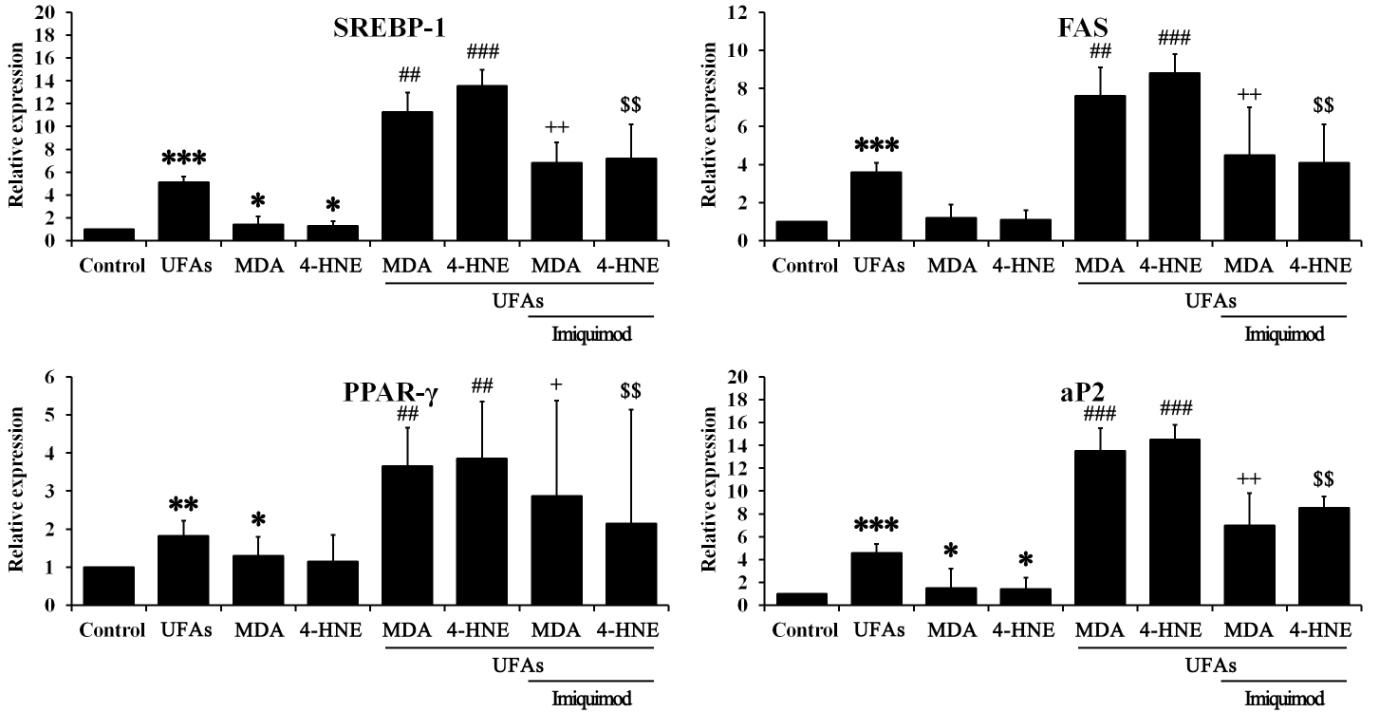


**Fig. S7. MDA and 4-HNE exacerbated progression of NAFLD via blockade of TLR7**

Normal mice were injected with or without 0.4 mg/kg MDA, 0.8 mg/kg 4-HNE, and 0.1 mg/kg imiquimod twice a week while eating a UFAs diet or a normal research diet for 8 weeks. Gene expression of SREBP-1, FAS, PPAR-γ, and aP2 in liver tissues was examined by real-time RT-PCR. Data are mean and SEM values (*n*=3). **p*<0.05 vs. untreated control mice. ***p*<0.01 vs. untreated control mice. ****p*<0.001 vs. untreated control mice. *^#^p*<0.05 vs. UFAs group. *^##^p*<0.01 vs. UFAs group. *^###^p*<0.001 vs. UFAs group. *^+^p*<0.05 vs. MDA + UFAs group. *^++^p*<0.01 vs. MDA + UFAs group. *^+++^p*<0.001 vs. MDA + UFAs group. *^$^p*<0.05 vs. 4-HNE + UFAs group. *^$$^p*<0.01 vs. 4-HNE + UFAs group. *^$$$^p*<0.001 vs. 4-HNE + UFAs group.


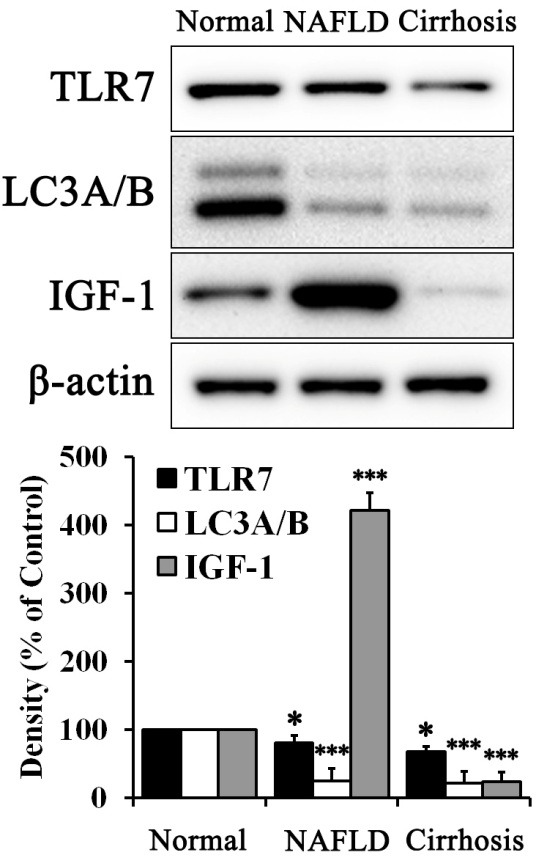


**Fig. S8. Comparative analysis of TLR7, LC3A/B, and IGF-1 protein expression in each stage of chronic liver disease**

Normal mice were fed a UFAs diet or a normal research diet for 8 weeks. Liver cirrhosis was induced by feeding mice a UFAs diet for 40 weeks. We performed immunoblotting to evaluate the protein expression of TLR7, LC3A/B, and IGF-1. Data are mean and SEM values (*n*=3). **p*<0.05 vs. untreated control. ****p*<0.001 vs. untreated control.


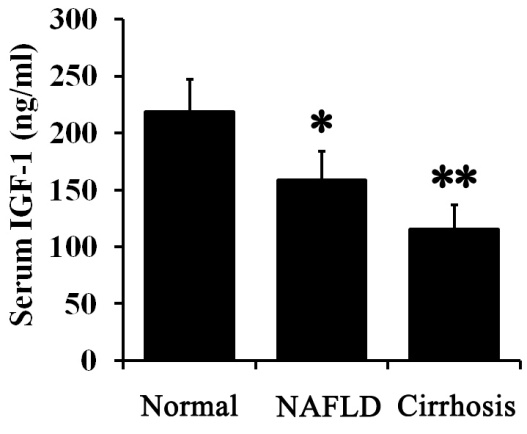


**Fig. S9. Comparative analysis of IGF-1 contents in serum from each stage of chronic liver disease**

Normal mice were fed a UFAs diet or a normal research diet for 8 weeks. We induced liver cirrhosis by feeding mice a UFAs diet for 40 weeks. We performed an ELISA for IGF-1 using serum. Data are mean and SEM values (*n*=3). **p*<0.05 vs. untreated control. ***p*<0.01 vs. untreated control.
